# The relationship between SV2A levels, neural activity, and cognitive function in healthy humans: A [11C]UCB-J PET and fMRI study

**DOI:** 10.1162/imag_a_00190

**Published:** 2024-06-10

**Authors:** Ekaterina Shatalina, Ellis Chika Onwordi, Thomas Whitehurst, Alexander Whittington, Ayla Mansur, Atheeshaan Arumuham, Ben Statton, Alaine Berry, Tiago Reis Marques, Roger N. Gunn, Sridhar Natesan, Matthew M. Nour, Eugenii A. Rabiner, Matthew B. Wall, Oliver D. Howes

**Affiliations:** Department of Psychosis Studies, Institute of Psychiatry, Psychology and Neuroscience, King’s College London, London, United Kingdom; MRC Laboratory of Medical Science, Imperial College London, London, United Kingdom; Centre for Psychiatry and Mental Health, Wolfson Institute of Population Health, Queen Mary University of London, London, United Kingdom; East London NHS Foundation Trust, London, United Kingdom; Invicro, London, United Kingdom; South London and Maudsley NHS Foundation Trust, London, United Kingdom; Faculty of Medicine, Imperial College London, London, United Kingdom; Departement of Psychiatry, Oxford University, Oxford, United Kingdom; Max Planck UCL Centre for Computational Psychiatry and Ageing Research, University College London, London, United Kingdom

**Keywords:** set shifting, task switching, working memory, synaptic density, multimodal neuroimaging

## Abstract

Synaptic terminal density is thought to influence cognitive function and neural activity, yet its role in cognition has not been explored in healthy humans. We examined these relationships using [11C]UCB-J positron emission tomography (PET) and functional magnetic resonance imaging (fMRI) in 25 healthy adults performing cognitive function tasks in the scanner. We found a significant positive association between synaptic terminal density, indicated by [11C]UCB-J PET distribution volume ratio (DVRcs), and neural activity during task switching (PLS-CA, second canonical component,*r*= 0.63,*p*= 0.043) with the thalamus-putamen data positively contributing to this relationship (PLS-CA loading 0.679, exploratory Pearson’s correlation*r *= 0.42,*p*= 0.044, uncorrected). Furthermore, synaptic terminal density predicted switch cost (PLS-R, R^2^= 0.45, RMSE = 0.06,*p*= 0.022), with DVRcs negatively correlating with switch cost in key brain regions including the dorsolateral prefrontal cortex and posterior frontal cortex. Conversely, no significant relationships were observed between [11C]UCB-J DVRcs and neural activity or performance measures in the N-back working memory task, suggesting interindividual differences in synaptic terminal density may be more closely related to some cognitive functions and not others.

## 1 Introduction

Synapses are specialized neural junctions that are central to information processing in the brain. They perform computations including summation, inhibition, coincidence detection, denoising, temporal integration, and temporal filtering (Abbott & Nelson, 2000;Abbott & Regehr, 2004;Soltani & Wang, 2010). Given that synapses are sites of neuronal computation, it has been long hypothesised that synaptic density may determine the computational power of a neural network and thus has a key role in cognitive performance. There are several bodies of experimental evidence supporting this, most measuring synaptic density using electron microscopy or array tomography or using proxy measures such as protein and mRNA markers specific to synapses (Calhoun et al., 1996;Kay et al., 2013).

Findings in rodent models suggest that inducing synaptic degradation or enhancement in brain regions such as the prefrontal cortex and hippocampus correlates with differences in cognitive function. For example, pharmacological or environmental-driven increases in synaptic density are associated with improved memory and other cognitive functions, such as in healthy animals exposed to environmental enrichment (Petrosini et al., 2009;Saito et al., 1994;Shen et al., 2019;Wang et al., 2020), animals administered estrogen and estrogen receptor modulators (Silva et al., 2000;Velázquez-Zamora et al., 2012) and animals administered ghrelin (Diano et al., 2006).

In contrast, in animals administered NMDA antagonists such as phencyclidine or ketamine (Elsworth, Hajszan, et al., 2011;Elsworth, Morrow, et al., 2011;Hajszan et al., 2006;Marrs et al., 2005;Smith et al., 2011), or the NMDA agonist ibotenic acid (Martínez-Torres et al., 2021), and in those exposed to chronic stress (Liston et al., 2006), synaptic loss co-occurs with impairments in cognition. Importantly, evidence of direct relationships has also been reported, with synapse formation being specifically associated with memory storage in the cerebellum of rats that underwent eye-blink conditioning (Kleim et al., 2002) and working memory errors being shown to be negatively associated with hippocampal spine density in rats (Mahmmoud et al., 2015).

The development of [11C]UCB-J positron emission tomography (PET) provides an*in vivo*measure of synaptic vesicle protein 2A (SV2A), which is ubiquitously expressed in synaptic terminals (Finnema et al., 2016,^2018^). SV2A PET imaging has been widely used as a proxy biomarker of presynaptic density (Finnema et al., 2016;Holmes et al., 2019;Onwordi et al., 2020,^2021^;Toyonaga et al., 2019). A preliminary study carried out in a cohort of 15 aged non-human primates found that working memory scores measured during a spatial-delayed response task were positively correlated with [11C]UCB-J binding in the dorsolateral prefrontal cortex (DLPFC) and hippocampus (Fang et al., 2020). Further research has been carried out in neuropsychiatric and neurodegenerative conditions where synaptic loss is considered a pathophysiological feature (Asch et al., 2022;Holmes et al., 2023;Howes et al., 2023;Mecca et al., 2022;Serrano et al., 2022). These studies show positive relationships between SV2A levels and measures of episodic and verbal memory, language, executive function, processing speed, visuospatial ability and global cognition measures in mild cognitive impairment (MCI) and Alzheimer’s disease studies (Fang et al., 2020;Mecca et al., 2022;O’Dell et al., 2021). They also show positive relationships between SV2A levels and verbal memory in post-traumatic stress disorder (PTSD), major depression, and cannabis use disorder (D’Souza et al., 2021;Holmes et al., 2019), along with negative relationships with response time on a visual attention task in individuals with a psychiatric comorbidity (Asch et al., 2022).

Furthermore, to date, out of four published studies investigating the link between synaptic density and brain function (Adams et al., 2023;Coomans et al., 2021;Fang et al., 2023;Holmes et al., 2019), two also evaluated cognition (Adams et al., 2023;Coomans et al., 2021). One study was carried out in post-traumatic stress disorder (PTSD) and major depressive disorder (MDD) participants and reported that SV2A levels in the DLPFC were negatively correlated with both resting state DLPFC-posterior cingulate cortex connectivity measured using fMRI and with performance during a working memory task performed outside of the scanner (Holmes et al., 2019). The second study used magnetoencephalography in participants with progressive supranuclear palsy and linked reductions in synaptic density in the inferior frontal cortex with altered superficial and granular layer glutamatergic excitation, which was predictive of individual differences in cognition (Adams et al., 2023). Thus far, no studies have looked at the link with brain function when subjects were performing cognitive tasks. One suitable method for testing such relationships would be by measuring the blood oxygen level dependent (BOLD) response using functional MRI, as the measure has a similar spatial resolution to [11C]UCB-J PET (Constable, 2023). BOLD FMRI is also thought to reflect synaptic firing, thus providing a measure of neural activity and whole-brain function that has a synaptic origin (Goense & Logothetis, 2008;Logothetis, 2007,^2008^;Logothetis & Wandell, 2004;Logothetis et al., 2001).

Overall, both preclinical findings and studies in humans with neuropsychiatric disorders indicate that synaptic markers are linked to cognitive function, and with neural activity. However, it remains unknown if synaptic markers are associated with cognitive performance in healthy humans or with neural activity when performing cognitive tasks. To determine this, we aimed to test two main hypotheses in healthy subjects based on the findings above: (1) that synaptic terminal density, as measured by [11C]UCB-J, is positively related to the fMRI response in task-relevant regions and (2) that synaptic terminal density in these regions is positively related to task performance. To test these, we used two tasks that engage different executive functions of the brain: the N-back working memory task and a task-switching exercise, which engages cognitive flexibility.

## 2 Methods

### 2.1 Approvals and recruitment

The study was approved by the West London & GTAC Research Ethics Committee (16/LO/1941) and was conducted in compliance with the principles of Good Clinical Practice (GCP), the Declaration of Helsinki (1996 Version), the Research Governance Framework for Health & Social Care, and the Administration of Radioactive Substances Advisory Committee (ARSAC) guidelines. Standard MRI screening procedures were followed and written informed consent was obtained from all participants.

Inclusion criteria were age 18 to 65 years old, having adequate command of English, and a normal blood coagulation test. Exclusion criteria were drug or alcohol dependence (except nicotine), physical illness, past or present neurological illness, pregnancy or lactating mothers, imaging contraindications, taking a drug known to interact with SV2A (including levetiracetam, brivaracetam, loratadine or quinine) (Danish et al., 2017) and current or past psychiatric diagnosis or family history of schizophrenia.

### 2.2 Magnetic resonance imaging acquisition

Data were acquired on a Siemens MAGNETOM Prisma 3 Tesla (3T) MRI scanner (Siemens Healthineers, Erlangen, Germany) with the in-built body coil used for radio frequency (RF) excitation and the manufacturer’s 64 channel phased-array head/neck coil for reception.

Whole-head anatomical T1-weighted images were acquired at the beginning of each scanning session using the Magnetisation Prepared Rapid Gradient Echo (MPRAGE) sequence, with parameters based on Alzheimer’s Disease Research Network (ADNI-GO); FOV 256 x 256 mm, 1 mm isotropic voxels, 176 sagittal slices, repetition time (TR) = 2300 ms, echo time (TE) = 2.98 ms, inversion time = 900 ms, flip angle 9°, bandwidth 200 Hz/pixel, Parallel Imaging factor (PI) of 2 (Jack Jr et al., 2008). All structural images were inspected by an experienced clinical neuroradiologist for unexpected findings of clinical significance. If such findings were identified, participants and their general practice teams were informed to facilitate follow-up, and they were excluded from the study.

Functional MRI data were acquired for a duration of nine minutes for each cognitive task using blood oxygen level dependent (BOLD) contrast, which measures neural activation in response to changes in cognitive load (e.g., during a task state). The sequence consisted of T2* weighted transverse echo planar image (EPI) slices. For the N-back working memory task, each run consisted of 270 volumes, collected in an ascending direction with a FoV = 192 x 192 mm, 3 mm isotropic voxels, 42 axial slices, TR = 2000 ms, TE = 20 ms, flip angle 62º, echo spacing = 0.71 ms, bandwidth = 1594 Hz/Px, PI = 2. For task switching, each run consisted of 180 volumes, collected in an ascending direction with a FOV = 250 x 250 mm, 2 x 2 x 3 mm voxel dimensions, 44 axial slices, TR = 3000 ms, TE = 30 ms, flip angle = 90º, echo spacing = 0.71 ms, bandwidth 1594 Hz/px, PI = 2.

### 2.3 N-back working memory task

**Fig. 1.**
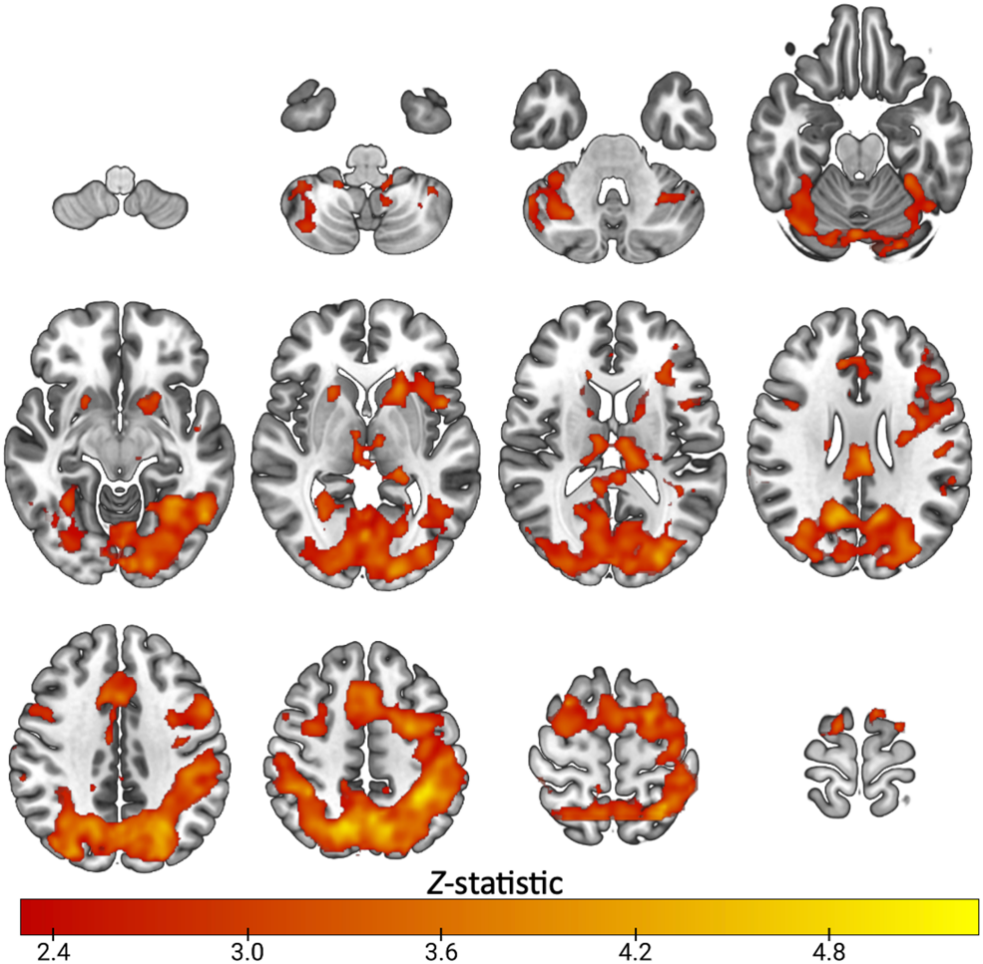
Mean activation during task switching in healthy subjects (n=23): Z. (Gaussianised T/F) statistical images representative of significant activation during the highSwitch>no-switch condition of the switching task. Images were thresholded using clusters determined by Z > 2.3 and a (corrected) cluster significance threshold of*p*= 0.05. Axial slices shown in MNI152 are: -52 -42 -32 -22-12; -2 8 18 28; 38 48 56 66

Participants completed a block-design N-back task designed to engage working memory. The task was adapted fromRagland et al. (2002). It was coded in PsychoPy version 2 (https://www.psychopy.org) and consisted of 0-back, 1-back, and 2-back blocks (Ragland et al., 2002) similar to that used previously and has been shown to produce reliable activation of the frontoparietal network (Demetriou et al., 2018;Nour et al., 2019). During the 0-back block, participants were required to remember an initial target letter and identify whether the subsequent letters matched the target. During the 1- and 2-back blocks, the participants had to recall whether the subsequent letters matched the letter presented one or two trials prior, respectively. The task is summarised inSupplementary Figure 1. Participants responded using an MRI-compatible response box (Nordic Neuro Labs, Bergen Norway) with the thumb and index fingers of their right hand (indicating ‘yes’ or ‘no,’ respectively). Each block lasted 20 seconds in total, containing 10 two-second trials, and was followed by a 10-second rest period. Six repetitions of each block were presented in a pseudorandomised order for a total task time of nine minutes. The percentage of correct responses out of the total responses was calculated for each block. Subjects who performed >2.5 SD lower than the group mean were excluded from the final analyses due to inadequate performance suggestive of non-engagement with the task.

### 2.4 Task switching task design

Participants completed an event-related switching task based on a task used previously (Kimberg et al., 2000). This task was selected as it engages a different executive function of the brain to the N-back working memory task. Specifically, the task engages cognitive flexibility which allows switching from one task to another (Cristofori et al., 2019). As summarized inSupplementary Figure 3, pairs of numbers and letters appear on the screen in blue or green text. If the number-letter pair appeared in green, participants were required to focus on the letter and respond if the letter is a vowel or consonant. If the number-letter pair appeared in blue, participants were required to focus on the number and respond if it is odd or even. All participants confirmed they do not have colour blindness. The task contained switching trials, defined as a trial in which the colour changed from the previous trial, and no-switch trials, where the colour remained the same as that of the previous trial. Each instance of switching/not switching was modelled as a single event, with 108 no-switch trials and 42 switch trials in total. Switch cost (the difference in reaction time between switch and no-switch conditions) was calculated as the main measure of task performance (Wylie & Allport, 2000). For both tasks, participants were trained on the task prior to the fMRI session and confirmed they remembered the rules when in the scanner.

### 2.5 Data preprocessing

All functional data and anatomical data were processed with FSL (FMRIB Software Library v5.0.4;http://www.fmrib.ox.ac.uk/fsl/). Brain Extraction Tool (BET) was used for brain extraction of the anatomical data and the fsl_anat script was used for additional anatomical data preprocessing. Motion correction was performed with FMRIB Linear Image Registration Tool (MCFLIRT), with spatial smoothing using a Gaussian kernel of full width at half maximum (FWHM) of 6 mm. Temporal high-pass filtering was applied with a 100 second cut-off threshold. A two-step co-registration was carried out, first registering functional data to the subject’s individual anatomical image and then to an anatomical template image in standard stereotactic space (MNI152). High motion for a subject was defined as a mean relative root-mean-square displacement that exceeded 0.5 mm, which measures displacement between subsequent images in the time series (Power et al., 2014). For subjects with high motion, plots of their mean and relative displacement were visually inspected, and if high motion affected over 30 consecutive volumes, they were excluded from the analyses.

### 2.6 First-level analyses and group-mean effect of tasks

First-level (subject-level) analyses, which model the effects of the task, were carried out in FSL’s FEAT module using the general linear model and FMRIB’s Improved Linear Model (FILM) pre-whitening. For the N-back task, the general linear model (GLM) design matrix comprised events for 0-back, 1-back, and 2-back conditions as explanatory regressors and with six standard head motion regressors as nuisance regressors. For task switching, the design matrix comprised separate explanatory regressors for switch and no-switch trials and six standard head motion regressors as nuisance regressors.

For both tasks, explanatory regressors (but not nuisance regressors) were convolved with a standard gamma haemodynamic response function (HRF) (SD = 3s, mean lag = 6s), with added temporal derivative and temporal filtering to match the pre-processing steps applied to the data (Aguirre et al., 1998). Contrasts were computed to model the effects of the working memory (1&2-back>0-back) and switching (switch>no-switch) for the N-back and switching tasks, respectively.

In second-level (group) analyses, we computed the mean effect of the cognitive load of the task across all subjects using FSL’s FLAME-1 module, with the 1&2-back>0-back contrast used for the N-back and the switch>no-switch contrast for task switching. Statistical significance of group-level activation patterns was determined using a whole-brain cluster-corrected significance threshold (significance threshold of Z = 2.3, cluster defining threshold of*p*<0.05) (Eklund et al., 2016) and identified regions of the brain that were significantly engaged by each task.

### 2.7 Generation of regions of interest

Regions of interest (ROIs) were defined*a priori*based on an automated coordinate-based meta-analysis via neurosynth.org using the terms ‘working memory’ (data from 1091 studies) and ‘switching’ (data from 193 studies) for the N-back and task switching analyses, respectively. Neurosynth automatically extracts activation coordinates from published studies tagged with a particular term and transforms these coordinates into the same stereotactic space for meta-analysis. A false-discovery-rate correction of 0.05 is then applied to identify differential activation for studies that include a term versus studies that do not include a term (Yarkoni et al., 2011). The activation map was separated into six task-relevant regions for each task that had no spatial overlap. For task switching, these were DLPFC, posterior frontal cortex, insula, ACC, parietal-precuneus and thalamus-putamen (Supplementary Fig. 4). For the N-back, these were the prefrontal cortex, dorsolateral prefrontal cortex (DLPFC), posterior dorsolateral frontal cortex, anterior cingulate cortex (ACC), parietal cortex and insula (Supplementary Fig. 2). In addition to the six neurosynth ROIs for the N-back, an additional hippocampal ROI was used for exploratory analyses only (i.e. not included in the partial least squares omnibus analyses described inSection 2.9below). The ROI for the bilateral hippocampus was derived from the CIC atlas (Tziortzi et al., 2011). While the hippocampus is not part of the working memory network and is not activated by the N-back task (Fig. 1andhttps://neurosynth.org/analyses/terms/working%20memory/), several previous studies investigating relationships between synaptic density and working memory include the hippocampus as an ROI (Fang et al., 2020;Holmes et al., 2019;Mahmmoud et al., 2015). Thus, to test if clinical and animal findings in this region translate to healthy humans, we chose to include it as an additional region of interest.

All ROI standard-space masks were binarized and back-projected into individual subject space, and parameter estimate values were extracted for each ROI mask image, and for each individual subject, using FSL’s featquery module.

### 2.8 [11C]UCB-J data acquisition and processing

[11C]UCB-J data were acquired as described previously byOnwordi et al. (2020)on a HiRez 6 PET/computed tomography scanner (Siemens Healthcare, Erlangen, Germany). [11C]UCB-J was administered as an intravenous injection over 20 seconds into the cubital vein and data were collected continuously for 90 min (26 frames: 8 × 15 s, 3 × 60 s, 5 × 120 s, 5 × 300 s and 5 × 600 s). Arterial blood samples were collected throughout the scan via a unilaterally placed arterial line to measure the arterial input function that included the associated processing of discrete samples to determine the plasma-to-whole blood ratio and the parent fraction.

Data were processed and modelled using MIAKAT (https://nmmitools.org/2019/01/01/miakat/). MIAKAT is implemented using MATLAB (version R2017a; The MathWorks, Inc., Natick, Mass.) and makes use of the FMRIB Software Library (version 4.1.9) functions for brain extraction, and SPM12 (http://www.fil.ion.ucl.ac.uk/spm) functions for image segmentation and registration.

Individual PET frames were corrected for radioactive decay and for head motion using rigid-body co-registration with the 16th frame used as the reference image. The T1-weighted MR image was co-registered to the summed PET image, after brain extraction using the Brain Extraction Tool (Smith, 2002).

Analysis was carried out using the 1-tissue compartment (1TC) tissue model using metabolite-corrected plasma input fraction with a fixed 5% blood volume correction, as this has been previously validated for this tracer (Finnema et al., 2018;Mansur et al., 2020). All voxel time-activity curves were analysed in subject space to derive a distribution volume (V_T_; mL/cm^3^) for each voxel, similar to previous work using this tracer (Mertens et al., 2020). Non-linear deformation parameters were derived by the diffeomorphic anatomical registration through exponentiated lie algebra (DARTEL) algorithm and used for the mapping of the T1 image into stereotaxic space, to transform each subject’s PET data into standard MNI152 space following kinetic modelling (Ashburner, 2007).

To calculate the main outcome measure, distribution volume ratio (DVRcs), a mask of the centrum semiovale (CS) region, was used to extract an average CS V_T_value for each subject. Following this, each subject’s parametric map was divided by their CS V_T_, to produce a parametric DVRcs map. DVRcs has been validated as an optimal outcome measure for this tracer in previous work (Mansur et al., 2020;Mertens et al., 2020). From the parametric maps, average DVRcs values were derived for each ROI. ROIs used were identical to those used for fMRI analysis and are described above. Variability of [11C]UCB-J DVRcs for each ROI was calculated using variance (square of the standard deviation) and coefficient of variation (ratio of standard deviation to the mean).

### 2.9 Statistical analyses

#### 2.9.1 Relationship between [11C]-UCB-J DVR and task-evoked neural activation

Our primary hypothesis concerns the statistical association between the spatial distribution of neural activation during executive function (measured with fMRI) and synaptic terminal density (measured with [11C]UCB-J PET). Both fMRI and PET measures comprise multiple variables (ROIs) per participant, necessitating a statistical approach capable of identifying robust statistical relationships between two multivariate datasets. For this reason, we used Partial Least Squares Canonical Analysis (PLS-CA) for each task to investigate the multivariate relationship between PET and fMRI datasets across the six ROIs (i.e., for each modality, a [number_participants, number_ROIs] matrix was computed). This method creates linear projections of the fMRI and PET data, each represented as a [n_participants, n_ROI] matrix. In each new multidimensional direction (*i*), the projected fMRI and PET datasets, termed ‘*scores*_*i*_’, show the highest possible covariance. Additionally, it ensures that scores in different projection directions remain orthogonal. This approach is suitable for neuroimaging data which often contain numerous inter-correlated variables (Mihalik et al., 2022).

PLS-CA was performed using the ‘PLSCanonical’ function from the ‘sklearn.cross_decomposition’ module in Python (Pedregosa et al., 2011). We opted for a two-component solution (i.e., identifying solutions which yielded projections of fMRI and PET datasets onto a two-dimensional subspace), allowing us to investigate the first and second most correlated latent structures between the two imaging modalities.

To determine the statistical significance of the identified PET-fMRI relationships (canonical correlations), we used a permutation testing approach, which offers a robust mechanism to derive*p*-values by comparing the observed canonical correlations against a null distribution of correlations obtained from randomly permuting the rows of the fMRI [n_participants, n_ROI] matrix multiple times, while holding the rows of the PET [n_participants, n_ROI] matrix fixed. In the present study, we used 1000 permutations for each PLS-CA, one run for each task, and applied a significance level of*p*<0.05 (i.e., the permutation-derived two-tailed*p*-value is the proportion of permutations that generate absolute correlation coefficients greater than the observed correlation coefficient, and an observed correlation is deemed statistically significant if the absolute value of this correlation is greater than the 950th largest absolute value of the empirical null distribution). An advantage of permutation testing is that it does not make parametric assumptions about underlying data, and enables a direct test of significance of an examined relationship between two variable sets (here, fMRI and PET) against an empirical null distribution that preserves all other statistical features of the data.

In addition to reporting canonical correlations, we also report canonical weights for each latent component, where these weights (a [1, number_ROI] vector for both fMRI and PET) signify the contribution of each original feature (from each ROI) to the canonical variates. This allows us to understand which brain regions primarily drive the shared information between the PET and fMRI data.

To investigate the nature of the relationships at the individual ROI level, we then conducted exploratory univariate analyses to investigate the PET-fMRI relationships in each ROI separately. These included Pearson’s correlation coefficients to assess relationships between [11C]UCB-J DVR and task fMRI and performance measures. p-Values were uncorrected for exploratory analyses and results are discussed as significant if*p*<0.05.

#### 2.9.2 Relationships between [11C]UCB-J DVRcs and task performance

For investigating relationships between PET and task performance measures, a Partial Least Squares Regression (PLS-R) was chosen as it is well suited for neuroimaging applications where multicollinearity in the predictor variables might be a concern, as is the case with [11C]UCB-J data. In this methodological framework, both the PET data and task performance metrics are projected onto a new set of orthogonal components. The objective is to maximize the covariance between these components, thus teasing out the latent structures in the data that are most relevant to the relationship between synaptic terminal density and cognitive performance. We used a two-component solution for the PLS-R.

The quality of our model’s predictions was quantified using the R^2^statistic and the root mean square error (RMSE). As described above, a permutation test was incorporated (1000 permutations), providing a non-parametric assessment of the model’s R^2^values. Exploratory univariate analyses were used to investigate the PET-fMRI relationships in each ROI separately. These included Pearson’s correlation coefficients to assess relationships between [11C]UCB-J DVR and task performance measures, with a significance threshold of*p*<0.05 (uncorrected).

## 3 Results

### 3.1 Demographic details and mean task switching activity

A total of 27 participants were recruited and completed the study. In total, 25 participants were included for N-back analyses (two excluded due to high motion, none due to non-performance) and 23 participants were included for task switching analyses (three excluded due to non-performance, one due to high motion). Data for switch cost for one subject were not available due to an MRI controller issue, the subject confirmed they understood the task once out of the scanner and their switch>no-switch first-level analyses showed engagement of relevant regions, so they were included in the analysis. Full sample details are presented inTable 1.

**Table 1.** Demographic details.

	Mean ± standard deviation for each fMRI task	
	Task switching	N-back
Sample size	n= 23	n= 25
Age (years)	34.52 ± 12.88	35.36 ± 12.46
Age range (years)	20-59	20-59
Female (n)	2	3
Weight (kg)	80.07 ± 13.40	82.57 ± 14.79
BMI (kg/m ^2^ )	25.58 ± 3.85	26.56 ± 4.20
Injected radioactivity (MBq)	260.58 ± 28.04	259.34 ± 27.35
Plasma fraction	0.24 ± 0.02	0.25 ± 0.03

Task switching widely engaged cortical regions across subjects, shown inFigure 1, with significant task-related activity spanning the dorsolateral prefrontal cortex, anterior cingulate cortex, posterior cingulate, insular cortex, thalamus, caudate, putamen, parietal, occipital and cerebellar regions (Z>2.3, cluster*p*<0.05). These regions overlapped with preselected ROIs (Supplementary Fig. 4).Supplementary Figure 5shows that selected ROIs had positive parameter estimates, indicating they were engaged by the task.

### 3.2 Task switching BOLD response and [11C]UCB-J DVRcs

As shown inFigure 2, we found that in the first canonical component, which captures the greatest amount of covariance in our data, there was no significant relationship between [11C]UCB-J DVRcs and switch>no-switch parameter estimates (Cov = 1.25,*r*= 0.28,*p*= 0.389). However, there was a statistically significant relationship between [11C]UCB-J DVRcs and switch>no-switch PE for the second canonical component (Cov = 0.25,*r*= 0.63,*p*= 0.043). The weights provide insights into which ROIs contribute most to these relationships and are presented inSupplementary Table 1. The thalamus-putamen ROI PET data contributed most to canonical variate 2 (weight 0.679). For the fMRI data, the posterior frontal cortex parameter estimates contributed most to the latent structure, where the weight was negative (–0.603), indicating an inverse relationship between the posterior frontal cortex high-switch>no-switch parameter estimates and the latent variable’s value. Exploratory analyses for these two regions are presented inFigure 3and show that there was a significant positive correlation between [11C]UCB-J DVRcs and high-switch>no-switch PE in the thalamus-putamen ROI (Pearson’s correlation,*r *= 0.42,*p*= 0.043, uncorrected,Fig. 3A), but no significant association between [11C]UCB-J DVRcs and high-switch>no-switch PE in the posterior frontal cortex ROI (Pearson’s correlation,*r *= 0.13,*p*= 0.556, uncorrected,Fig. 3B). Additional exploratory analyses for remaining ROIs did not identify any significant relationships and are included inSupplementary Figure 6.

**Fig. 2.**
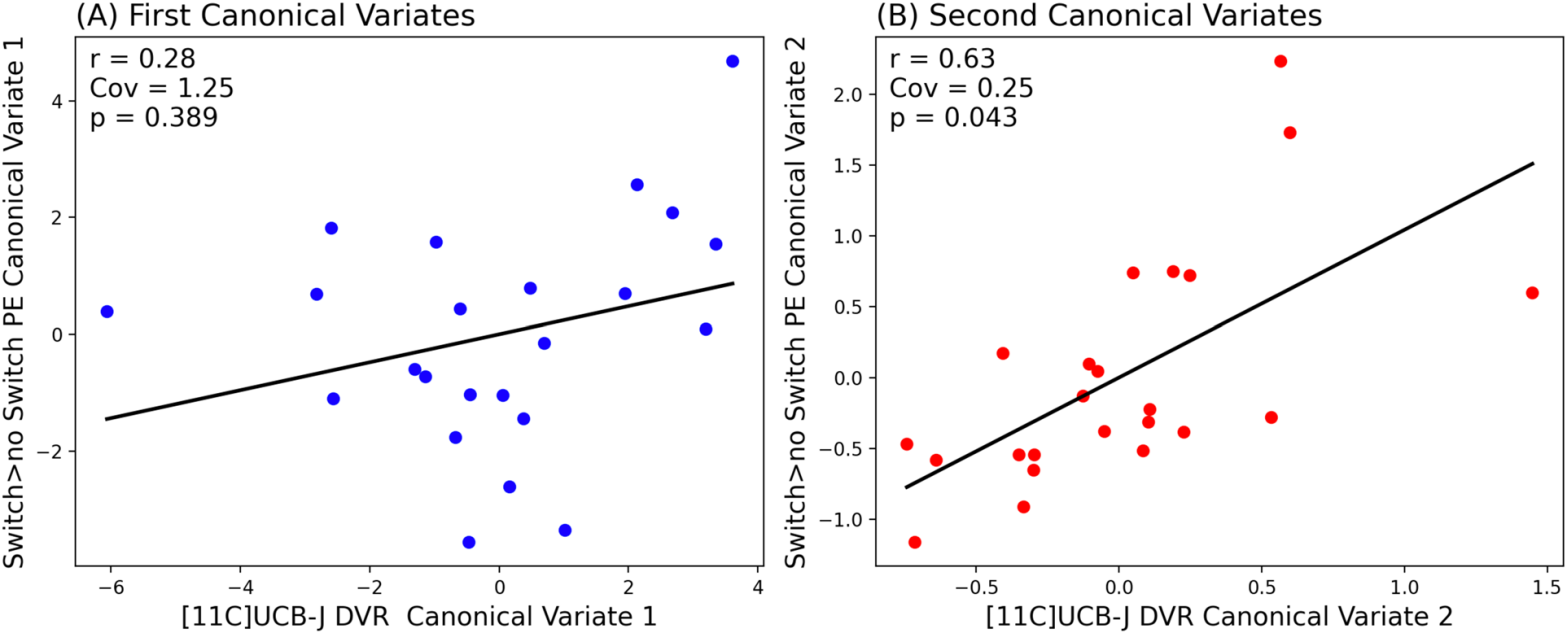
Partial Least Squares Canonical Analysis (PLS-CA) showing a strong positive correlation between [11C]UCB-J DVRcs and task switching-related neural activity in the second canonical component (B) and no significant relationship in the first canonical component (A). Cov— covariance captured by each canonical correlation, significance threshold*p*<0.05.

**Fig. 3.**
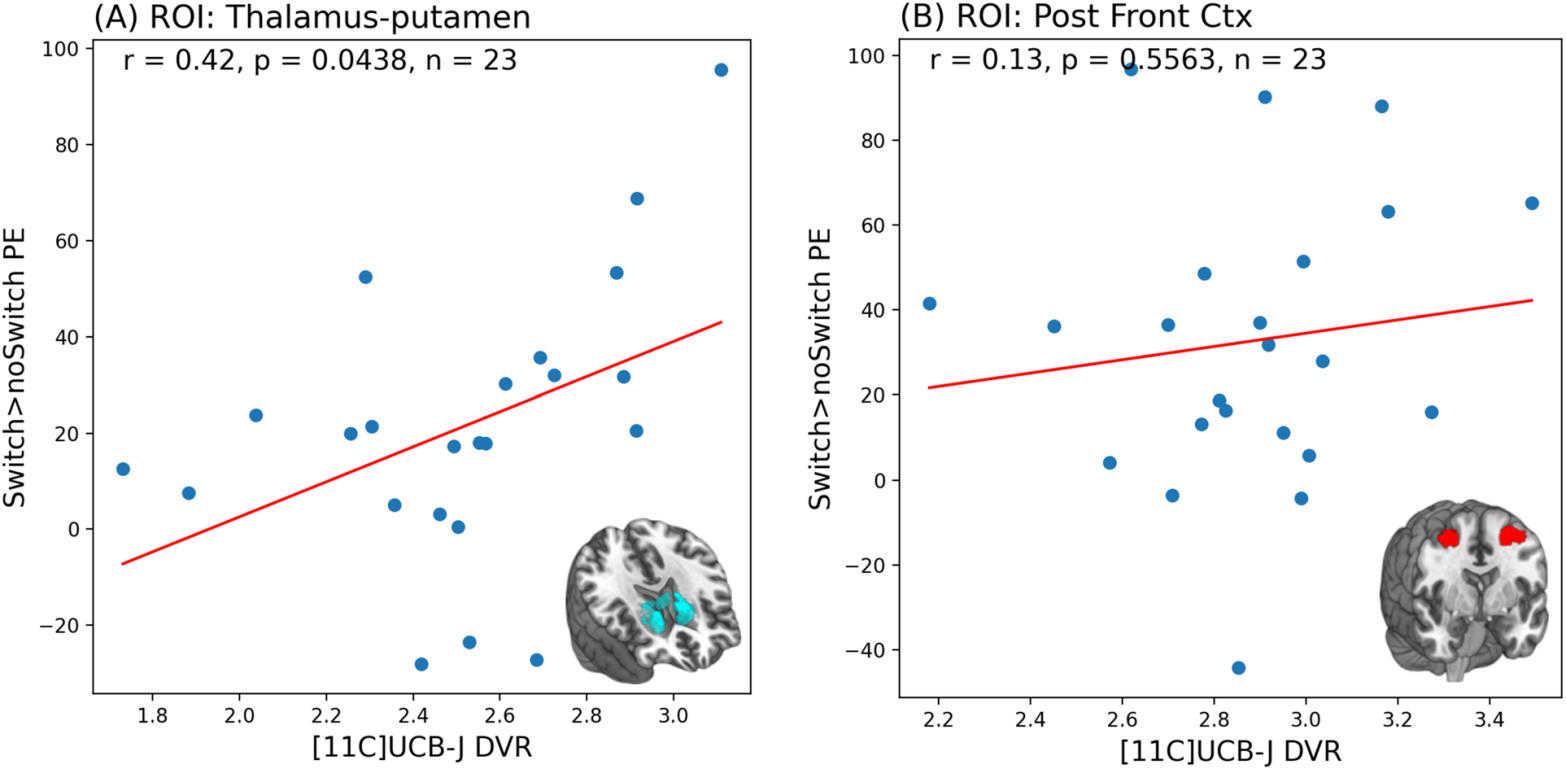
Scatter plot and Pearson’s correlation results showing a significant positive relationship between [11C]UCB-J DVRcs and high-switch>no-switch parameter estimates (PE) in the thalamus-putamen region of interest (ROI) (A), and lack of significant relationships between [11C]UCB-J DVRcs and high-switch>no-switch PE in the posterior frontal cortex ROI (B)

### 3.3 Switch cost and [11C]UCB-J DVRcs

As shown inFigure 4, [11C]UCB-J DVRcs values from six task-relevant regions of interest were significantly predictive of switch cost (PLS-regression, R^2^= 0.45, RMSE = 0.06,*p*= 0.022), accounting for 45% of the variance in the switch cost data. For the PET data in the first component (covariance captured -0.105), the parietal-precuneus ROI had the highest weight (0.485), closely followed by the posterior cortex ROI (0.460). In the second component (covariance captured 0.014), for the PET data, the thalamus-putamen ROI was the most prominent with a weight of 0.5438 (supplementary table 2). Exploratory analyses, presented infigure 5, showed significant negative correlations between [11C]UCB-J DVRcs and switch cost in the insula (*r*=–0.45,*p*= 0.034, uncorrected), DLPFC (*r*=–0.52,*p*= 0.013, uncorrected), parietal precuneus (*r*=–0.59,*p*= 0.004, uncorrected), posterior frontal cortex (*r*=–0.56,*p*= 0.007, uncorrected) and ACC (*r*=–0.42,*p*= 0.049, uncorrected) and a trend towards negative relationships in the thalamus-putamen ROI (*r*= 0.39,*p*= 0.073, uncorrected).

**Fig. 4.**
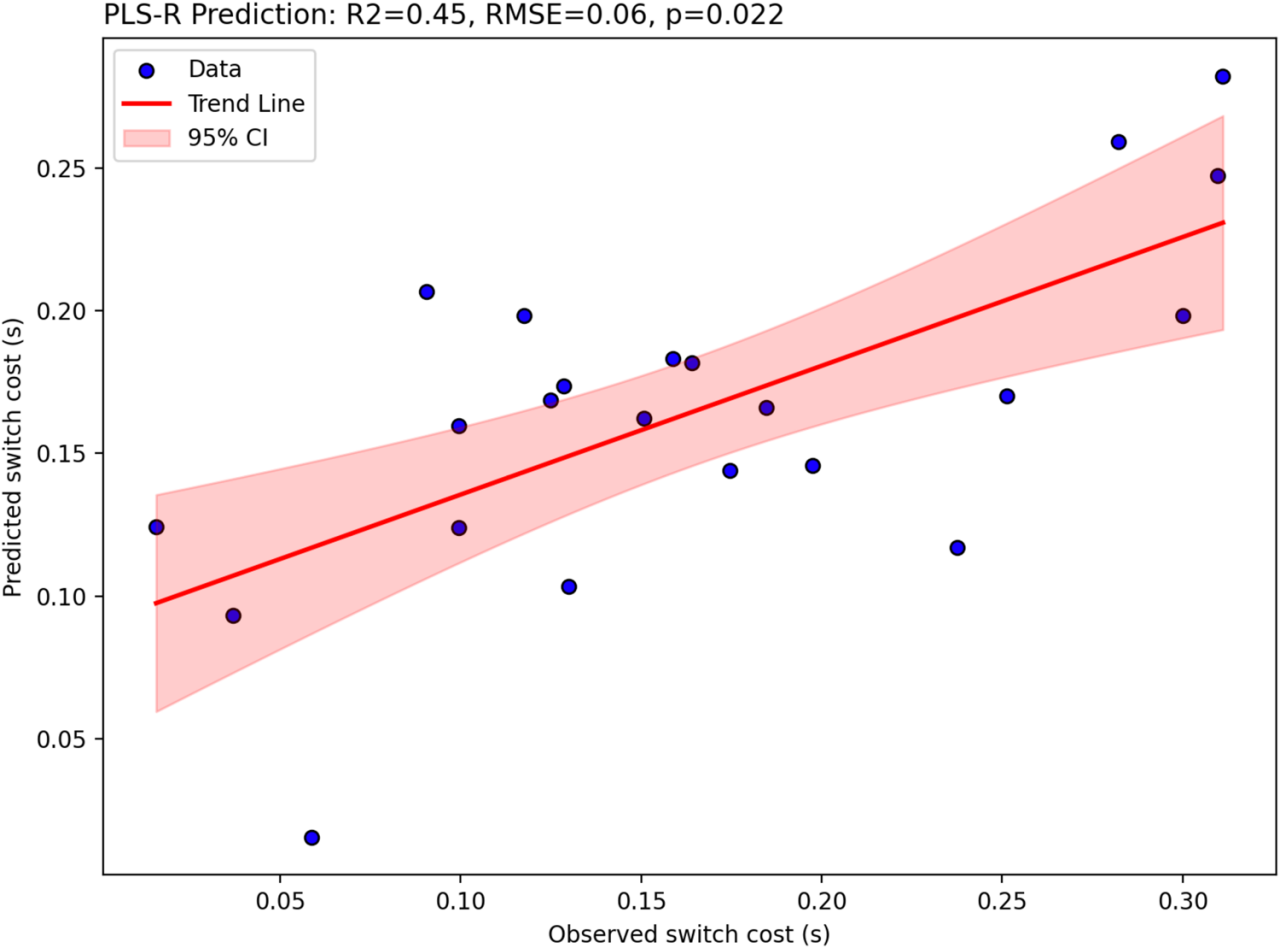
Two-component partial-least-squares regression results showing switch cost (s) are significantly predicted by [11C]UCB-J DVRcs values across six task-switching relevant regions of interest. Figure shows predicted switch cost plotted against observed switch cost, trend-line in red and 95% confidence interval shaded red.

**Fig. 5.**
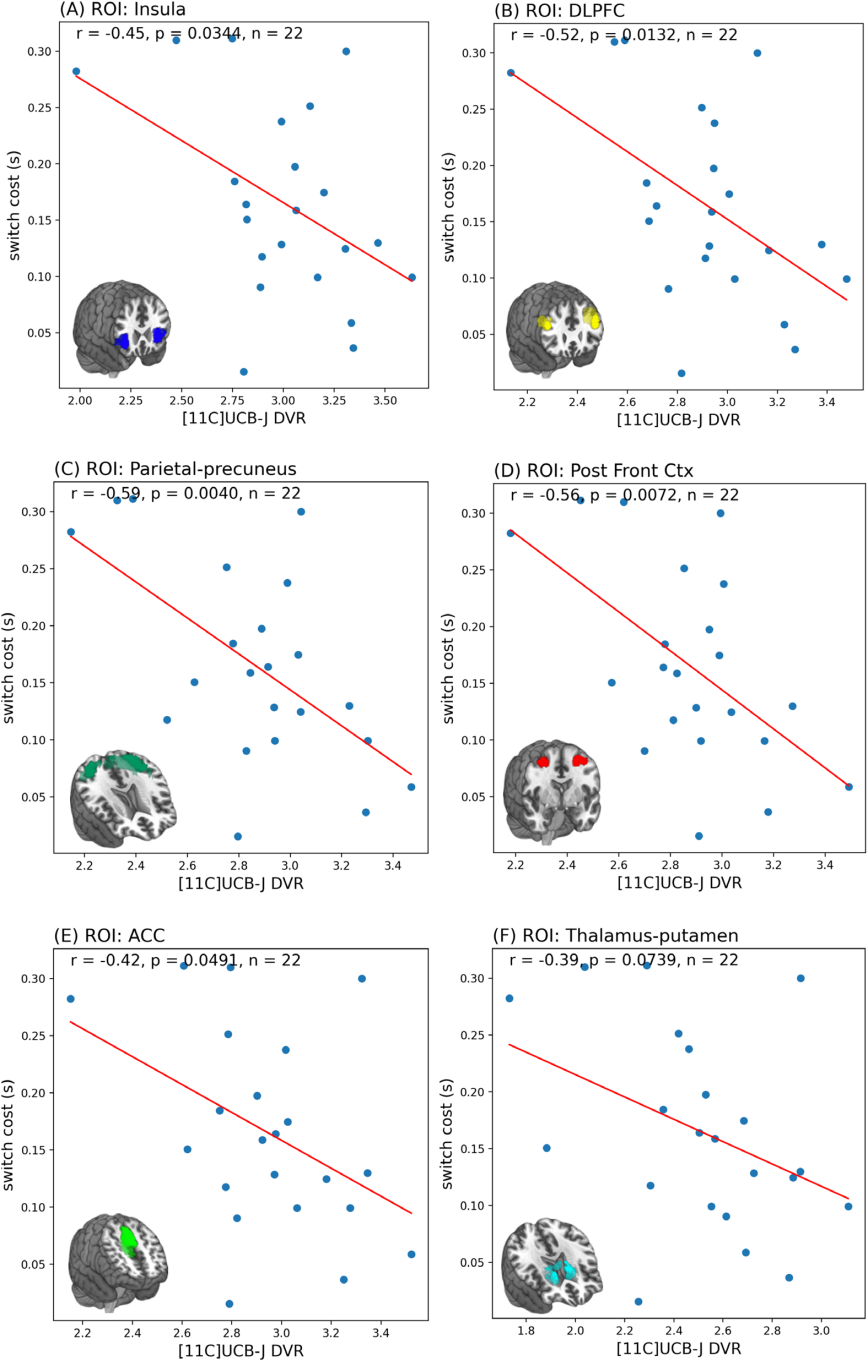
Scatter plots showing results of Pearson’s correlations that show significant negative correlations between switch cost and [11C]UCB-J DVRcs in the (A) Insula, (B) dorsolateral prefrontal cortex (DLPFC), (C) parietal-precuneus, (D) posterior frontal cortex, (E) anterior cingulate cortex, and a trend towards a negative correlation in the (F) thalamus-putamen regions of interest.

### 3.4 N-back working memory task

As shown inFigure 6, the N-back task engaged prefrontal cortical regions, middle frontal gyrus, dorsolateral prefrontal cortex, precentral gyrus, right insula, parietal, occipital and temporal cortex, and cerebellum. These regions overlapped with the preselected ROIs (Supplementary Fig. 2), and had positive parameter estimate values as shown inSupplementary Figure 7, indicating task engagement. With respect to [11C]UCB-J–N-back relationships,Figure 7shows that neither the first nor the second canonical components in the PLS-CA analysis were significantly correlated, which suggests that there was no relationship between [11C]UCB-J DVRcs and working memory-induced neural activity in our sample of participants (PLS-CA, first pair of components*r*= 0.23,*p*= 0.697, Cov = 1.02, second components*r*= 0.40,*p*= 0.647, Cov = 0.16, exploratory analyses shown inSupplementary Fig. 8). The canonical weights showing how the fMRI and PET data contributed to the latent variables are shown inSupplementary Table 3.Figure 8shows that [11C]UCB-J DVRcs values across the six predefined working memory ROIs were not predictive of N-back behavioural task performance (PLS-R, R^2^= 0.11, RMSE = 0.09,*p*= 0.782), with weights for PET values of each ROI contributing to the latent space provided inSupplementary Table 4. Exploratory analyses presented inSupplementary Figure 9confirm that there were no significant relationships between N-back task performance and [11C]UCB-J DVRcs in any of the six neurosynth-derived ROIs (Pearson’s correlations, all*p*>0.05, uncorrected). There were also no significant relationships between N-back neural activity and [11C]UCB-J DVRcs or between N-back task performance and [11C]UCB-J DVRcs in the hippocampus (Pearson’s*r*= 0.01,*p*= 0.969, uncorrected and*r*= 0.27,*p*= 0.196, uncorrected respectively,Supplementary Fig. 10). Finally, across subjects, [11C]UCB-J DVRcs had low variability in all ROIs used for both tasks, with analyses presented inSupplementary Table 5.

**Fig. 6.**
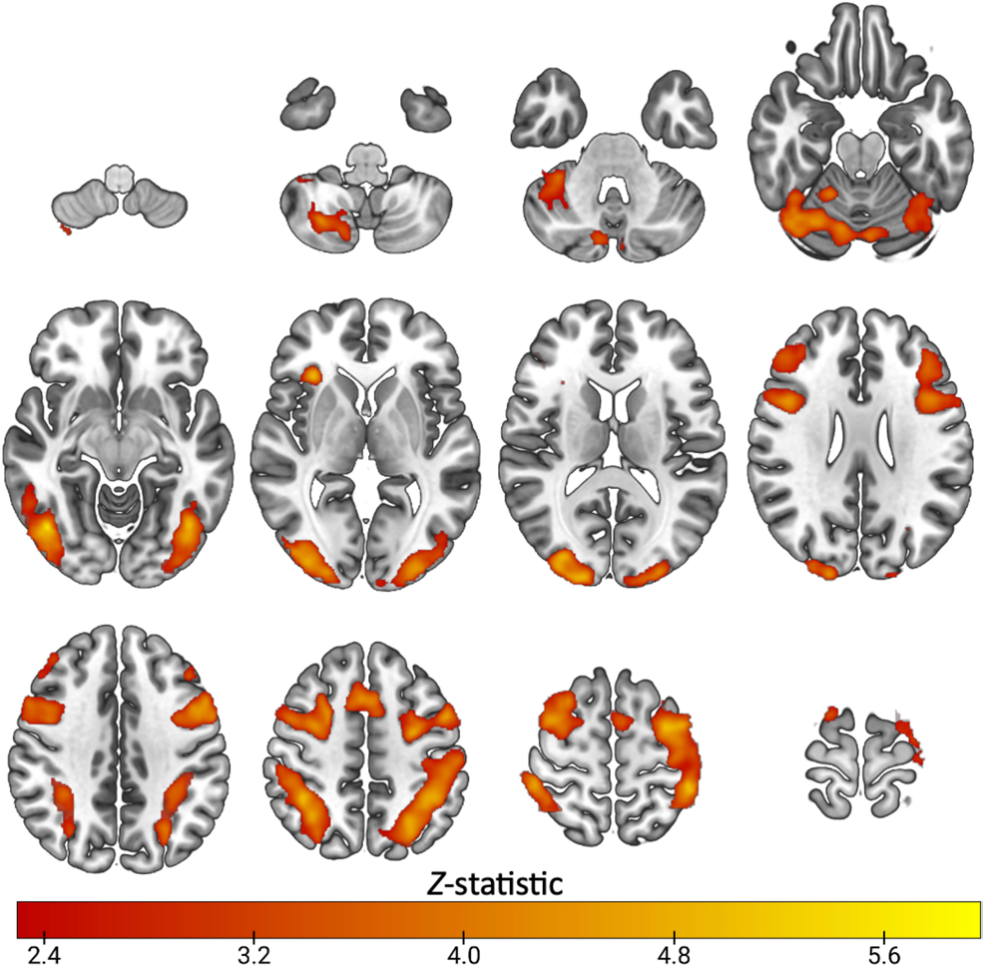
N-back mean effect of task in the working memory contrast (*n*=25): Z (Gaussianised T/F) statistical images representative of significant activation during the 1&2back>0back condition of the N-back working memory task. Images were thresholded using clusters determined by Z > 2.3 and a (corrected) cluster significance threshold of*p*= 0.05, axial slices shown in MNI152 are -52 -42 -32 -22-12; -2 8 18 28; 38 48 56 66

**Fig. 7.**
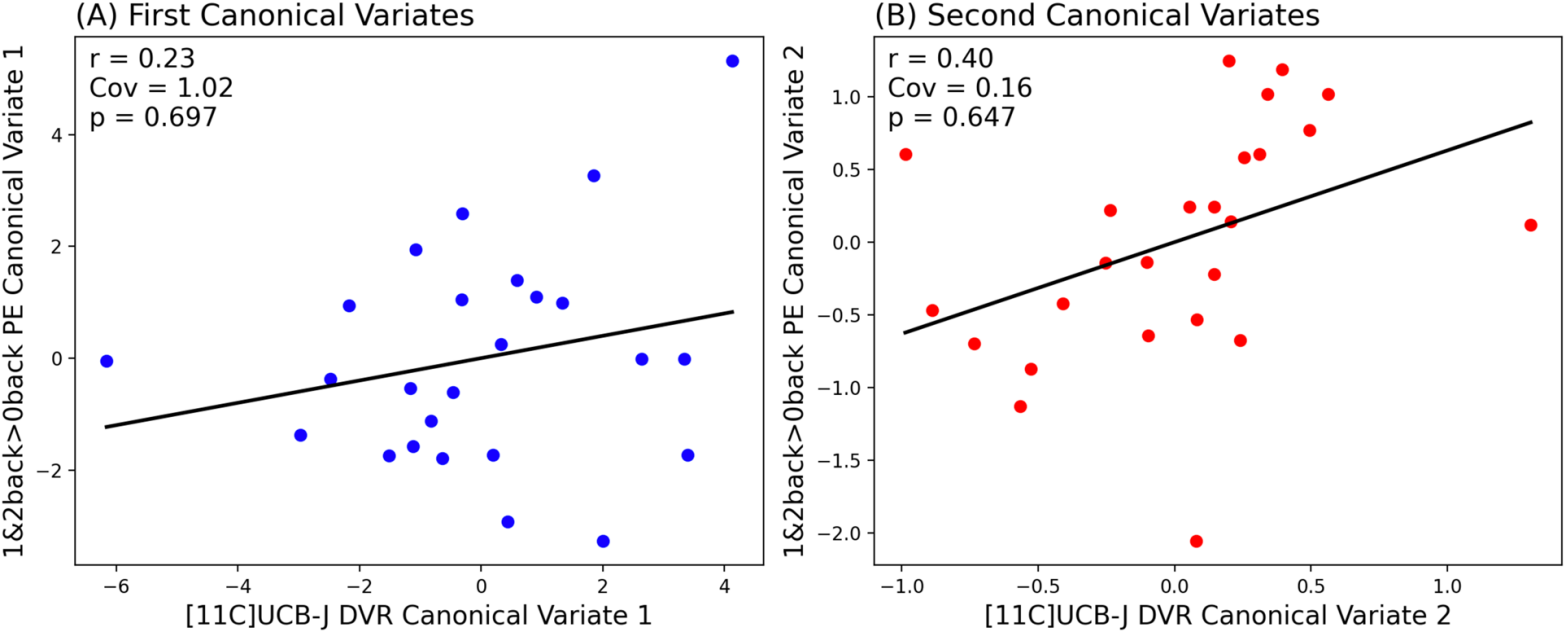
Partial Least Squares Canonical Analysis (PLS-CA) showing no significant correlation between [11C]UCB-J DVRcs and working memory-related neural activity (1&2back>0back PE) in both the first (A) and second (B) canonical components. Cov – covariance captured by each pair of components, significance threshold*p*<0.05.

**Fig. 8.**
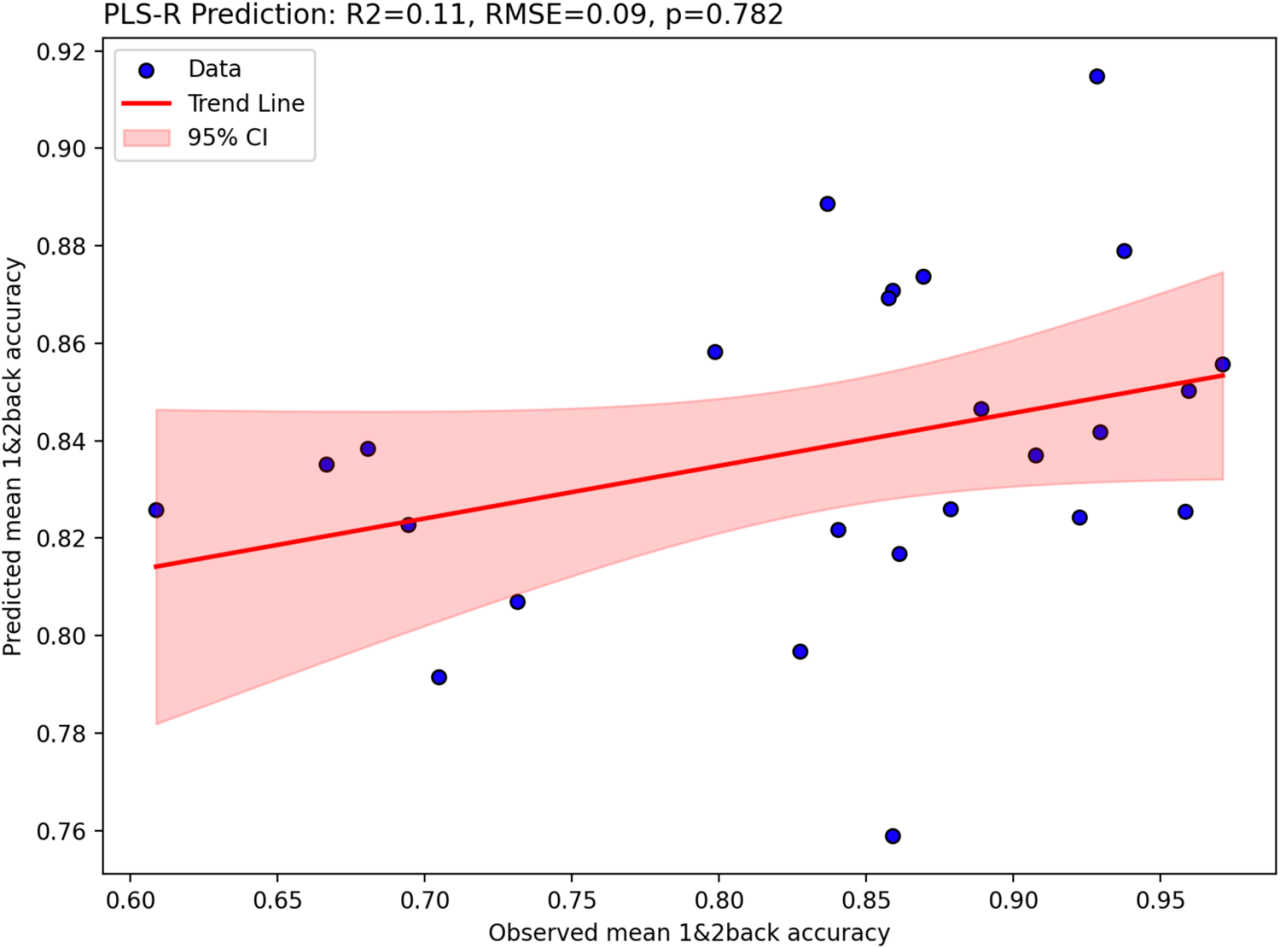
Two-component partial-least-squares regression results showing that N-back (s) is not significantly predicted by [11C]UCB-J DVR values across 6 working memory-relevant regions of interest. Figure shows predicted mean 1- and 2-back accuracy plotted against observed mean 1- and 2-back accuracy, trend-line in red, and 95% confidence interval shaded red.

## 4 Discussion

Our results show, for the first time in healthy humans, a relationship between [11C]UCB-J DVRcs and cognitive task-related neural activity and behavioral performance. This extends a prior report that [11C]UCB-J binding is related to resting state fMRI activity (Fang et al., 2023). Our exploratory analyses showed that [11C]UCB-J DVRcs in the region that contributed most to this relationship, the thalamus-putamen ROI, was significantly positively correlated with switching-related activation of this region. We also found that [11C]UCB-J DVRcs was associated with behavioural switch cost, with negative correlations in the parietal-precuneus, posterior frontal cortex, insula, DLPFC and ACC regions, and, at a trend level, in the thalamus-putamen. As shorter switch cost time indicates better performance, these findings indicate that higher [11C]UCB-J DVRcs is associated with better performance.

In contrast to task switching, we found no relationships between [11C]UCB-J DVRcs and either task performance or neural activity in the N-back working memory task. Our finding extends a prior report of a lack of relationship between SV2A levels and working memory performance in a joint cohort of individuals with MDD and PTSD (Holmes et al., 2019), to show this in a non-clinical population sample. One preclinical study did report negative correlations between working memory errors from the radial arm maze paradigm and spine density in hippocampal CA1 and CA3 basal dendrites of rats (Mahmmoud et al., 2015). However, this paradigm may not translate to verbal working memory measured in humans, which would explain this discrepancy between our findings that show no relationships between N-back task performance and synaptic terminal density in the hippocampus and with previous work byHolmes et al. (2019).

### 4.1 Interpretation of findings

[11C]UCB-J DVRcs is a measure of specific tracer binding to the SV2A protein, shown to have high test–retest reliability (Tuncel et al., 2021,^2022^). Given that SV2A is ubiquitously expressed in synaptic vesicles across the brain and its levels are correlated with those of synaptophysin, the gold-standard molecular synaptic density marker, [11C]UCB-J DVRcs can be interpreted as a measure of synaptic terminal density (Finnema et al., 2016). Thus, our data indicate that higher synaptic terminal density is related to neural activity, as indexed by BOLD response during task switching, and with better task performance. This extends preclinical findings linking cognitive performance with synaptic density (Kleim et al., 2002;Mahmmoud et al., 2015) and, importantly, extends findings of [11C]UCB-J imaging studies in clinical populations showing that cognitive performance is also linked with synaptic terminal density in healthy humans.

A key finding from our present study is that synaptic terminal density is related to task switching neural response and task performance, but not to working memory. One reason for this might be that switching is more closely related to processing speed (Mukherjee et al., 2021). For example, higher synaptic density is thought to enable quicker transitions between network states, increasing the efficiency of switching between tasks (Herd et al., 2014). In contrast, working memory accuracy reflects temporary information storage in a defined array of synapses and may rely on persistent activity, rather than the density of synapses (Compte et al., 2000;Lim & Goldman, 2013;Lundqvist et al., 2018). Thus, these processes have different synaptic demands, with greater synaptic density facilitating the rapid recruitment of new circuits or faster suppression of previously active ones.

### 4.2 Strengths and limitations

One key strength of this study is the joint use of BOLD fMRI and [11C]UCB-J PET in the same subjects. Firstly, this allows for extracting measures relating to synaptic terminal density and neural activity from identical regions in each subject. Secondly, BOLD fMRI has been shown to reflect local field potentials, which reflect synaptic firing (Herreras, 2016;Logothetis, 2007;Logothetis & Wandell, 2004;Logothetis et al., 2001;Viswanathan & Freeman, 2007), making both the structural and functional measures in this study specific to synapses.

There are also several considerations relevant to the findings presented in this study. While SV2A levels have lower variability than those of synaptophysin, which is typically used as a synaptic density marker in vitro (Mutch et al., 2011), it is currently unknown if there are significant interindividual differences in the expression of SV2A per vesicle or differences in vesicle number per synapse, and whether these may be contributing to differences in [11C]UCB-J binding. Further research into the SV2A protein and synaptic vesicle variability in health and disease would provide valuable confirmation that these factors do not introduce variance to [11C]UCB-J signal. Furthermore, [11C]UCB-J imaging does not provide information about the types of synapses present in each region. Given that interneurons and glutamatergic synapses have different metabolic demands (Sotero & Trujillo-Barreto, 2007), one reason we might not see the relationship between working memory task performance/activity and synaptic terminal density is because the different synapse types contribute to BOLD in a variable manner, and individual differences in network organisation may be more closely related to working memory performance. In addition, [11C]UCB-J PET and other PET approaches are limited by a low spatial resolution of around 3 mm, which means that each voxel may contain millions of synapses belonging to different cell types. Therefore, it is possible that very subtle patterns of interindividual variability in synaptic density are not accurately captured by this imaging approach, but might still contribute to functional differences.

One other key consideration for interpreting our findings is whether the present study had the power to detect weak correlations between fMRI measures and measures of SV2A density. While the sample size is reasonable for human PET studies and provides >80% power to detect moderate or stronger correlations (*r*>0.5), it may be underpowered to detect weaker correlations. For example, to detect a correlation of >0.2 would require upwards of 47 subjects, calculated based on (Hulley et al., 2001). Thus, the findings presented do not exclude the possibility of weaker relationships. However, the clinical significance of weaker relationships is questionable as they would explain less than 10% of the variance in measures. In addition to this, our sample was collected as part of a larger study of synaptic terminal density in schizophrenia and participants were recruited to match the ages and sexes of the clinical cohort (Onwordi et al., 2020,^2024^). Due to this, few female participants (~10%) were included, which raises the importance of replicating these findings in a larger mixed cohort to see if these findings may generalise. Furthermore, relationships between neural activity and [11C]UCB-J DVRcs were absent in some ROIs and in the first component of the PLS-CA, which indicates these relationships are highly complex. This places importance on replicating these findings in future studies.

The final consideration is that while we have been able to identify relationships between synaptic terminal density, fMRI response and switch cost, we are not able to draw conclusions about the causal mechanisms linking these parameters. To understand the causal link between synaptic terminal density and switch cost, experiments where synaptic terminal density is dynamically modulated and switch cost is measured should be incorporated into future studies.

### 4.3 Implications and future directions

The main implication of our study is that individual differences in synaptic terminal density could explain differences in performance and brain activity in some cognitive tasks, but not others. This has implications for normal ageing, where synaptic terminal density has been shown to gradually decline (Mansur et al., 2020;Toyonaga et al., 2019), as our findings raise the question whether certain executive functions of the brain may be more vulnerable to synaptic loss than others.

Targeting synaptic terminal loss may also be a promising biological target for preventing age-related decline in cognitive function and for disorders where synaptic loss and cognitive decline are key pathophysiological features, such as Alzheimer’s disease. Thus, further work should focus on clinical populations and on studies where synaptic terminal density may be dynamically modulated. In addition to this, our data suggest that switch cost should be explored as a phenotypic marker of synaptic terminal density in future work.

Finally, our findings contribute to the body of literature addressing questions around how variability in synaptic density contributes to differences in brain function in health and disease (Adams et al., 2023;Coomans et al., 2021;Fang et al., 2023;Holmes et al., 2019). In our present study, we focused on BOLD contrast magnitude, due to its specificity to synaptic firing. However, future work investigating the link between synaptic terminal density and other measures of brain function such as functional or effective connectivity has potential to advance our understanding of these questions.

## 5 Conclusions

Together our findings suggest that presynaptic terminal density is related to both switch cost and task switching-induced neural activation, but not with task performance and task-induced neural activation during working memory. These findings suggest that synaptic terminal density may be important for some, but not all, cognitive tasks.

## Supplementary Material

Supplementary Material

## Data Availability

The datasets used and/or analysed during the current study are available from the corresponding author upon reasonable request.

## References

[b1] Abbott , L. , & Regehr , W. G. ( 2004 ). Synaptic computation . Nature , 431 ( 7010 ), 796 – 803 . 10.1038/nature03010 15483601

[b2] Abbott , L. F. , & Nelson , S. B. ( 2000 ). Synaptic plasticity: Taming the beast . Nature Neuroscience , 3 ( 11 ), 1178 – 1183 . 10.1038/81453 11127835

[b3] Adams , N. E. , Jafarian , A. , Perry , A. , Rouse , M. A. , Shaw , A. D. , Murley , A. G. , Cope , T. E. , Bevan-Jones , W. R. , Passamonti , L. , & Street , D. ( 2023 ). Neurophysiological consequences of synapse loss in progressive supranuclear palsy . Brain , 146 ( 6 ), 2584 – 2594 . 10.1093/brain/awac471 36514918 PMC10232290

[b4] Aguirre , G. K. , Zarahn , E. , & D’Esposito , M. ( 1998 ). The variability of human, BOLD hemodynamic responses . Neuroimage , 8 ( 4 ), 360 – 369 . 10.1006/nimg.1998.0369 9811554

[b5] Asch , R. H. , Holmes , S. E. , Jastreboff , A. M. , Potenza , M. N. , Baldassarri , S. R. , Carson , R. E. , Pietrzak , R. H. , & Esterlis , I. ( 2022 ). Lower synaptic density is associated with psychiatric and cognitive alterations in obesity . Neuropsychopharmacology , 47 ( 2 ), 543 – 552 . 10.1038/s41386-021-01111-5 34294874 PMC8674236

[b6] Ashburner , J. ( 2007 ). A fast diffeomorphic image registration algorithm . Neuroimage , 38 ( 1 ), 95 – 113 . 10.1016/j.neuroimage.2007.07.007 17761438

[b7] Calhoun , M. E. , Jucker , M. , Martin , L. J. , Thinakaran , G. , Price , D. L. , & Mouton , P. R. ( 1996 ). Comparative evaluation of synaptophysin-based methods for quantification of synapses . Journal of Neurocytology , 25 ( 1 ), 821 – 828 . 10.1007/bf02284844 9023727

[b8] Compte , A. , Brunel , N. , Goldman-Rakic , P. S. , & Wang , X.-J. ( 2000 ). Synaptic mechanisms and network dynamics underlying spatial working memory in a cortical network model . Cerebral Cortex , 10 ( 9 ), 910 – 923 . 10.1093/cercor/10.9.910 10982751

[b9] Constable , R. T. ( 2023 ). Challenges in fMRI and its limitations . Functional Neuroradiology: Principles and Clinical Applications , 497 – 510 . 10.1007/978-3-031-10909-6_22

[b10] Coomans , E. M. , Schoonhoven , D. N. , Tuncel , H. , Verfaillie , S. C. , Wolters , E. E. , Boellaard , R. , Ossenkoppele , R. , den Braber , A. , Scheper , W. , & Schober , P. ( 2021 ). In vivo tau pathology is associated with synaptic loss and altered synaptic function . Alzheimer’s Research & Therapy , 13 ( 1 ), 1 – 13 . 10.1186/s13195-021-00772-0 PMC7866464 33546722

[b11] Cristofori , I. , Cohen-Zimerman , S. , & Grafman , J. ( 2019 ). Executive functions . Handbook of Clinical Neurology , 163 , 197 – 219 . 10.1016/b978-0-12-804281-6.00011-2 31590731

[b12] D’Souza , D. C. , Radhakrishnan , R. , Naganawa , M. , Ganesh , S. , Nabulsi , N. , Najafzadeh , S. , Ropchan , J. , Ranganathan , M. , Cortes-Briones , J. , & Huang , Y. ( 2021 ). Preliminary in vivo evidence of lower hippocampal synaptic density in cannabis use disorder . Molecular Psychiatry , 26 ( 7 ), 3192 – 3200 . 10.1038/s41380-020-00891-4 32973170

[b13] Danish , A. , Namasivayam , V. , Schiedel , A. C. , & Müller , C. E. ( 2017 ). Interaction of approved drugs with synaptic vesicle protein 2A . Archiv der Pharmazie , 350 ( 3–4 ), 1700003 . 10.1002/ardp.201700003 28220535

[b14] Demetriou , L. , Kowalczyk , O. S. , Tyson , G. , Bello , T. , Newbould , R. D. , & Wall , M. B. ( 2018 ). A comprehensive evaluation of increasing temporal resolution with multiband-accelerated protocols and effects on statistical outcome measures in fMRI . Neuroimage , 176 , 404 – 416 . 10.1016/j.neuroimage.2018.05.011 29738911

[b15] Diano , S. , Farr , S. A. , Benoit , S. C. , McNay , E. C. , Silva da , I., Horvath , B. , Gaskin , F. S. , Nonaka , N. , Jaeger , L. B. , & Banks , W. A. ( 2006 ). Ghrelin controls hippocampal spine synapse density and memory performance . Nature Neuroscience , 9 ( 3 ), 381 – 388 . 10.1038/nn1656 16491079

[b16] Eklund , A. , Nichols , T. E. , & Knutsson , H. ( 2016 ). Cluster failure: Why fMRI inferences for spatial extent have inflated false-positive rates . Proceedings of the National Academy of Sciences , 113 ( 28 ), 7900 – 7905 . 10.1073/pnas.1602413113 PMC4948312 27357684

[b17] Elsworth , J. D. , Hajszan , T. , Leranth , C. , & Roth , R. H. ( 2011 ). Loss of asymmetric spine synapses in dorsolateral prefrontal cortex of cognitively impaired phencyclidine-treated monkeys . International Journal of Neuropsychopharmacology , 14 ( 10 ), 1411 – 1415 . 10.1017/s1461145711000939 21733230 PMC3399728

[b18] Elsworth , J. D. , Morrow , B. A. , Hajszan , T. , Leranth , C. , & Roth , R. H. ( 2011 ). Phencyclidine-induced loss of asymmetric spine synapses in rodent prefrontal cortex is reversed by acute and chronic treatment with olanzapine . Neuropsychopharmacology , 36 ( 10 ), 2054 – 2061 . 10.1038/npp.2011.96 21677652 PMC3158322

[b20] Fang , X. T. , Volpi , T. , Holmes , S. E. , Esterlis , I. , Carson , R. E. , & Worhunsky , P. D. ( 2023 ). Linking resting-state network fluctuations with systems of coherent synaptic density: A multimodal fMRI and 11C-UCB-J PET study . Frontiers in Human Neuroscience , 17 , 1124254 . 10.3389/fnhum.2023.1124254 36908710 PMC9995441

[b21] Fang , X. T. , Williams , G. , Castner , S. , Holden , D. , Zheng , M. Q. , Najafzadeh , S. , Ropchan , J. R. , Arnsten , A. F. , Horvath , T. , & Carson , R. E. ( 2020 ). The aging rhesus macaque as a potential model for Alzheimer’s disease/dementia: An in vivo study of [11C] PIB,[11C] UCB‐j,[18F] MK‐6240 and working memory performance: Development of new models and analysis methods/validation of pre‐clinical methods . Alzheimer’s & Dementia , 16 , e038467 . 10.1002/alz.038467

[b22] Finnema , S. J. , Nabulsi , N. B. , Eid , T. , Detyniecki , K. , Lin , S.-f. , Chen , M.-K. , Dhaher , R. , Matuskey , D. , Baum , E. , & Holden , D. ( 2016 ). Imaging synaptic density in the living human brain . Science Translational Medicine , 8 ( 348 ), 348ra396 – 348ra396 . 10.1126/scitranslmed.aaf6667 27440727

[b23] Finnema , S. J. , Nabulsi , N. B. , Mercier , J. , Lin , S.-f. , Chen , M.-K. , Matuskey , D. , Gallezot , J.-D. , Henry , S. , Hannestad , J. , & Huang , Y. ( 2018 ). Kinetic evaluation and test–retest reproducibility of [11C] UCB-J, a novel radioligand for positron emission tomography imaging of synaptic vesicle glycoprotein 2A in humans . Journal of Cerebral Blood Flow & Metabolism , 38 ( 11 ), 2041 – 2052 . 10.1177/0271678x17724947 28792356 PMC6259313

[b24] Goense , J. B. , & Logothetis , N. K. ( 2008 ). Neurophysiology of the BOLD fMRI signal in awake monkeys . Current Biology , 18 ( 9 ), 631 – 640 . 10.1016/j.cub.2008.03.054 18439825

[b25] Hajszan , T. , Leranth , C. , & Roth , R. H. ( 2006 ). Subchronic phencyclidine treatment decreases the number of dendritic spine synapses in the rat prefrontal cortex . Biological Psychiatry , 60 ( 6 ), 639 – 644 . 10.1016/j.biopsych.2006.03.015 16814748

[b26] Herd , S. A. , Hazy , T. E. , Chatham , C. H. , Brant , A. M. , & Friedman , N. P. ( 2014 ). A neural network model of individual differences in task switching abilities . Neuropsychologia , 62 , 375 – 389 . 10.1016/j.neuropsychologia.2014.04.014 24791709 PMC4167201

[b27] Herreras , O. ( 2016 ). Local field potentials: Myths and misunderstandings . Frontiers in Neural Circuits , 10 , 101 . 10.3389/fncir.2016.00101 28018180 PMC5156830

[b28] Holmes , S. E. , Abdallah , C. , & Esterlis , I. ( 2023 ). Imaging synaptic density in depression . Neuropsychopharmacology , 48 ( 1 ), 186 – 190 . 10.1038/s41386-022-01368-4 35768568 PMC9700860

[b29] Holmes , S. E. , Scheinost , D. , Finnema , S. J. , Naganawa , M. , Davis , M. T. , DellaGioia , N. , Nabulsi , N. , Matuskey , D. , Angarita , G. A. , & Pietrzak , R. H. ( 2019 ). Lower synaptic density is associated with depression severity and network alterations . Nature Communications , 10 ( 1 ), 1 – 10 . 10.1038/s41467-019-09562-7 PMC6449365 30948709

[b30] Howes , O. D. , Cummings , C. , Chapman , G. E. , & Shatalina , E. ( 2023 ). Neuroimaging in schizophrenia: An overview of findings and their implications for synaptic changes . Neuropsychopharmacology , 48 ( 1 ), 151 – 167 . 10.1038/s41386-022-01426-x 36056106 PMC9700830

[b31] Hulley , S. B. , Cummings , S. R. , Browner , W. S. , Grady D. , & Newman , T. B. ( 2013 ). Estimating sample size and power: Applications and examples . In Designing clinical research: An epidemiologic approach ( 4th ed., pp. 55 – 83 ). Philadelphia, PA : Lippincott Williams & Wilkins .

[b32] Jack Jr , C. R. , Bernstein , M. A. , Fox , N. C. , Thompson , P. , Alexander , G. , Harvey , D. , Borowski , B. , Britson , P. J. , Whitwell , L. J. , & Ward , C. ( 2008 ). The Alzheimer’s disease neuroimaging initiative (ADNI): MRI methods . Journal of Magnetic Resonance Imaging: An Official Journal of the International Society for Magnetic Resonance in Medicine , 27 ( 4 ), 685 – 691 . 10.1002/jmri.21049 PMC2544629 18302232

[b33] Kay , K. R. , Smith , C. , Wright , A. K. , Serrano-Pozo , A. , Pooler , A. M. , Koffie , R. , Bastin , M. E. , Bak , T. H. , Abrahams , S. , & Kopeikina , K. J. ( 2013 ). Studying synapses in human brain with array tomography and electron microscopy . Nature Protocols , 8 ( 7 ), 1366 – 1380 . 10.1038/nprot.2013.078 23787894 PMC3712649

[b34] Kimberg , D. Y. , Aguirre , G. K. , & D’Esposito , M. ( 2000 ). Modulation of task-related neural activity in task-switching: an fMRI study . Cognitive Brain Research , 10 ( 1–2 ), 189 – 196 . 10.1016/s0926-6410(00)00016-1 10978708

[b35] Kleim , J. A. , Jr Freeman , H. J. , Bruneau , R. , Nolan , B. C. , Cooper , N. R. , Zook , A. , & Walters , D. ( 2002 ). Synapse formation is associated with memory storage in the cerebellum . Proceedings of the National Academy of Sciences , 99 ( 20 ), 13228 – 13231 . 10.1073/pnas.202483399 PMC130615 12235373

[b36] Lim , S. , & Goldman , M. S. ( 2013 ). Balanced cortical microcircuitry for maintaining information in working memory . Nature Neuroscience , 16 ( 9 ), 1306 – 1314 . 10.1038/nn.3492 23955560 PMC3772089

[b37] Liston , C. , Miller , M. M. , Goldwater , D. S. , Radley , J. J. , Rocher , A. B. , Hof , P. R. , Morrison , J. H. , & McEwen , B. S. ( 2006 ). Stress-induced alterations in prefrontal cortical dendritic morphology predict selective impairments in perceptual attentional set-shifting . Journal of Neuroscience , 26 ( 30 ), 7870 – 7874 . 10.1523/jneurosci.1184-06.2006 16870732 PMC6674229

[b38] Logothetis , N. K. ( 2007 ). The ins and outs of fMRI signals . Nature Neuroscience , 10 ( 10 ), 1230 – 1232 . 10.1038/nn1007-1230 17893716

[b39] Logothetis , N. K. ( 2008 ). What we can do and what we cannot do with fMRI . Nature , 453 ( 7197 ), 869 – 878 . 10.1038/nature06976 18548064

[b40] Logothetis , N. K. , Pauls , J. , Augath , M. , Trinath , T. , & Oeltermann , A. ( 2001 ). Neurophysiological investigation of the basis of the fMRI signal . Nature , 412 ( 6843 ), 150 – 157 . 10.1038/35084005 11449264

[b41] Logothetis , N. K. , & Wandell , B. A. ( 2004 ). Interpreting the BOLD signal . Annual Review of Physiology , 66 , 735 – 769 . 10.1146/annurev.physiol.66.082602.092845 14977420

[b42] Lundqvist , M. , Herman , P. , & Miller , E. K. ( 2018 ). Working memory: Delay activity, yes! Persistent activity? Maybe not . Journal of Neuroscience , 38 ( 32 ), 7013 – 7019 . 10.1523/jneurosci.2485-17.2018 30089640 PMC6083456

[b43] Mahmmoud , R. R. , Sase , S. , Aher , Y. D. , Sase , A. , Gröger , M. , Mokhtar , M. , Höger , H. , & Lubec , G. ( 2015 ). Spatial and working memory is linked to spine density and mushroom spines . PloS One , 10 ( 10 ), e0139739 . 10.1371/journal.pone.0139739 26469788 PMC4607435

[b44] Mansur , A. , Rabiner , E. A. , Comley , R. A. , Lewis , Y. , Middleton , L. T. , Huiban , M. , Passchier , J. , Tsukada , H. , & Gunn , R. N. ( 2020 ). Characterization of 3 PET tracers for quantification of mitochondrial and synaptic function in healthy human brain: 18F-BCPP-EF, 11C-SA-4503, and 11C-UCB-J . Journal of Nuclear Medicine , 61 ( 1 ), 96 – 103 . 10.2967/jnumed.119.228080 31324712

[b45] Marrs , W. , Kuperman , J. , Avedian , T. , Roth , R. H. , & Jentsch , J. D. ( 2005 ). Alpha-2 adrenoceptor activation inhibits phencyclidine-induced deficits of spatial working memory in rats . Neuropsychopharmacology , 30 ( 8 ), 1500 – 1510 . 10.1038/sj.npp.1300700 15714223

[b46] Martínez-Torres , N. I. , Vázquez-Hernández , N. , Martín-Amaya-Barajas , F. L. , Flores-Soto , M. , & González-Burgos , I. ( 2021 ). Ibotenic acid induced lesions impair the modulation of dendritic spine plasticity in the prefrontal cortex and amygdala, a phenomenon that underlies working memory and social behavior . European Journal of Pharmacology , 896 , 173883 . 10.1016/j.ejphar.2021.173883 33513334

[b47] Mecca , A. P. , O’Dell , R. S. , Sharp , E. S. , Banks , E. R. , Bartlett , H. H. , Zhao , W. , Lipior , S. , Diepenbrock , N. G. , Chen , M. K. , & Naganawa , M. ( 2022 ). Synaptic density and cognitive performance in Alzheimer’s disease: A PET imaging study with [11C] UCB‐J . Alzheimer’s & Dementia . 10.1002/alz.12582 PMC9381645 35174954

[b48] Mertens , N. , Maguire , R. P. , Serdons , K. , Lacroix , B. , Mercier , J. , Sciberras , D. , Van Laere , K. , & Koole , M. ( 2020 ). Validation of parametric methods for [11C] UCB-J PET imaging using subcortical white matter as reference tissue . Molecular Imaging and Biology , 22 ( 2 ), 444 – 452 . 10.1007/s11307-019-01387-6 31209780

[b49] Mihalik , A. , Chapman , J. , Adams , R. A. , Winter , N. R. , Ferreira , F. S. , Shawe-Taylor , J. , Mourão-Miranda , J. , & Alzheimer’s Disease Neuroimaging Initiative . ( 2022 ). Canonical correlation analysis and partial least squares for identifying brain-behaviour associations: A tutorial and a comparative study . Biological Psychiatry: Cognitive Neuroscience and Neuroimaging . 10.1016/j.bpsc.2022.07.012 35952973

[b50] Mukherjee , A. , Lam , N. H. , Wimmer , R. D. , & Halassa , M. M. ( 2021 ). Thalamic circuits for independent control of prefrontal signal and noise . Nature , 600 ( 7887 ), 100 – 104 . 10.1038/s41586-021-04056-3 34614503 PMC8636261

[b51] Mutch , S. A. , Kensel-Hammes , P. , Gadd , J. C. , Fujimoto , B. S. , Allen , R. W. , Schiro , P. G. , Lorenz , R. M. , Kuyper , C. L. , Kuo , J. S. , & Bajjalieh , S. M. ( 2011 ). Protein quantification at the single vesicle level reveals that a subset of synaptic vesicle proteins are trafficked with high precision . Journal of Neuroscience , 31 ( 4 ), 1461 – 1470 . 10.1523/jneurosci.3805-10.2011 21273430 PMC3078718

[b52] Nour , M. M. , Dahoun , T. , McCutcheon , R. A. , Adams , R. A. , Wall , M. B. , & Howes , O. D. ( 2019 ). Task-induced functional brain connectivity mediates the relationship between striatal D2/3 receptors and working memory . Elife , 8 , e45045 . 10.7554/elife.45045 31290741 PMC6620042

[b53] O’Dell , R. , Mecca , A. P. , Sharp , E. S. , Banks , E. R. , Bartlett , H. H. , Chen , M.-K. , Naganawa , M. , Toyonaga , T. , Harris , J. E. , & Ni , G. S. ( 2021 ). Synaptic loss is associated with cognitive impairment in early Alzheimer’s disease: A PET imaging study with [11C] UCB-J . Biological Psychiatry , 89 ( 9 ), S107 – S108 . 10.1016/j.biopsych.2021.02.276

[b54] Onwordi , E. C. , Halff , E. F. , Whitehurst , T. , Mansur , A. , Cotel , M.-C. , Wells , L. , Creeney , H. , Bonsall , D. , Rogdaki , M. , & Shatalina , E. ( 2020 ). Synaptic density marker SV2A is reduced in schizophrenia patients and unaffected by antipsychotics in rats . Nature Communications , 11 ( 1 ), 1 – 11 . 10.1038/s41467-019-14122-0 PMC6959348 31937764

[b55] Onwordi , E. C. , Whitehurst , T. , Mansur , A. , Statton , B. , Berry , A. , Quinlan , M. , O’Regan , D. P. , Rogdaki , M. , Marques , T. R. , & Rabiner , E. A. ( 2021 ). The relationship between synaptic density marker SV2A, glutamate and N-acetyl aspartate levels in healthy volunteers and schizophrenia: A multimodal PET and magnetic resonance spectroscopy brain imaging study . Translational Psychiatry , 11 ( 1 ), 1 – 9 . 10.1038/s41398-021-01515-3 34282130 PMC8290006

[b56] Onwordi , E. C. , Whitehurst , T. , Shatalina , E. , Mansur , A. , Arumuham , A. , Osugo , M. , Marques , T. R. , Jauhar , S. , Gupta , S. , & Mehrotra , R. ( 2024 ). Synaptic terminal density early in the course of schizophrenia: An in vivo UCB-J positron emission tomographic imaging study of SV2A . Biological Psychiatry , 95 ( 7 ), 639 – 646 . 10.1016/j.biopsych.2023.05.022 37330164 PMC10923626

[b57] Pedregosa , F. , Varoquaux , G. , Gramfort , A. , Michel , V. , Thirion , B. , Grisel , O. , Blondel , M. , Prettenhofer , P. , Weiss , R. , & Dubourg , V. ( 2011 ). Scikit-learn: Machine learning in Python . The Journal of Machine Learning Research , 12 , 2825 – 2830 . 10.3389/fninf.2014.00014

[b58] Petrosini , L. , De Bartolo , P. , Foti , F. , Gelfo , F. , Cutuli , D. , Leggio , M. G. , & Mandolesi , L. ( 2009 ). On whether the environmental enrichment may provide cognitive and brain reserves . Brain Research Reviews , 61 ( 2 ), 221 – 239 . 10.1016/j.brainresrev.2009.07.002 19631687

[b59] Power , J. D. , Mitra , A. , Laumann , T. O. , Snyder , A. Z. , Schlaggar , B. L. , & Petersen , S. E. ( 2014 ). Methods to detect, characterize, and remove motion artifact in resting state fMRI . Neuroimage , 84 , 320 – 341 . 10.1016/j.neuroimage.2013.08.048 23994314 PMC3849338

[b60] Ragland , J. D. , Turetsky , B. I. , Gur , R. C. , Gunning-Dixon , F. , Turner , T. , Schroeder , L. , Chan , R. , & Gur , R. E. ( 2002 ). Working memory for complex figures: An fMRI comparison of letter and fractal n-back tasks . Neuropsychology , 16 ( 3 ), 370 . 10.1037//0894-4105.16.3.370 12146684 PMC4332798

[b61] Saito , S. , Kobayashi , S. , Ohashi , Y. , Igarashi , M. , Komiya , Y. , & Ando , S. ( 1994 ). Decreased synaptic density in aged brains and its prevention by rearing under enriched environment as revealed by synaptophysin contents . Journal of Neuroscience Research , 39 ( 1 ), 57 – 62 . 10.1002/jnr.490390108 7807593

[b62] Serrano , M. E. , Kim , E. , Petrinovic , M. M. , Turkheimer , F. , & Cash , D. ( 2022 ). Imaging synaptic density: The next holy grail of neuroscience ? Frontiers in Neuroscience , 16 , 796129 . 10.3389/fnins.2022.796129 35401097 PMC8990757

[b63] Shen , J. , Li , Y. , Qu , C. , Xu , L. , Sun , H. , & Zhang , J. ( 2019 ). The enriched environment ameliorates chronic unpredictable mild stress-induced depressive-like behaviors and cognitive impairment by activating the SIRT1/miR-134 signaling pathway in hippocampus . Journal of Affective Disorders , 248 , 81 – 90 . 10.1016/j.jad.2019.01.031 30716615

[b64] Silva , I. , Mello , L. E. , Freymüller , E. , Haidar , M. A. , & Baracat , E. C. ( 2000 ). Estrogen, progestogen and tamoxifen increase synaptic density of the hippocampus of ovariectomized rats . Neuroscience Letters , 291 ( 3 ), 183 – 186 . 10.1016/s0304-3940(00)01410-5 10984637

[b65] Smith , J. W. , Gastambide , F. , Gilmour , G. , Dix , S. , Foss , J. , Lloyd , K. , Malik , N. , & Tricklebank , M. ( 2011 ). A comparison of the effects of ketamine and phencyclidine with other antagonists of the NMDA receptor in rodent assays of attention and working memory . Psychopharmacology , 217 ( 2 ), 255 – 269 . 10.1007/s00213-011-2277-5 21484239

[b66] Smith , S. M. ( 2002 ). Fast robust automated brain extraction . Human Brain Mapping , 17 ( 3 ), 143 – 155 . 10.1002/hbm.10062 12391568 PMC6871816

[b67] Soltani , A. , & Wang , X.-J. ( 2010 ). Synaptic computation underlying probabilistic inference . Nature Neuroscience , 13 ( 1 ), 112 – 119 . 10.1038/nn.2450 20010823 PMC2921378

[b68] Sotero , R. C. , & Trujillo-Barreto , N. J. ( 2007 ). Modelling the role of excitatory and inhibitory neuronal activity in the generation of the BOLD signal . Neuroimage , 35 ( 1 ), 149 – 165 . 10.1016/j.neuroimage.2006.10.027 17234435

[b80] Toyonaga , T. , Lu , Y. , Naganawa , M. , Matuskey , D. , Mecca , A. , Pittman , B. , Nabulsi , N. , Finnema , S. , Chen , M. K. , Malison , R. , & Esterlis , I. ( 2019 ). Relationship of Age and Synaptic Density: A 11C‑UCB‑J PET Study in Healthy Controls with Partial Volume Correction. https://jnm.snmjournals.org/content/60/supplement_1/423

[b70] Tuncel , H. , Boellaard , R. , Coomans , E. M. , de Vries , E. F. , Glaudemans , A. W. , Feltes , P. K. , García , D. V. , Verfaillie , S. C. , Wolters , E. E. , & Sweeney , S. P. ( 2021 ). Kinetics and 28-day test–retest repeatability and reproducibility of [11C] UCB-J PET brain imaging . Journal of Cerebral Blood Flow & Metabolism , 41 ( 6 ), 1338 – 1350 . 10.1177/0271678x20964248 34013797 PMC8138337

[b71] Tuncel , H. , Boellaard , R. , Coomans , E. M. , Hollander-Meeuwsen , M. d. , de Vries , E. F. , Glaudemans , A. W. , Feltes , P. K. , García , D. V. , Verfaillie , S. C. , & Wolters , E. E. ( 2022 ). Validation and test–retest repeatability performance of parametric methods for [11C] UCB-J PET . EJNMMI Research , 12 ( 1 ), 1 – 12 . 10.1186/s13550-021-00874-8 35072802 PMC8786991

[b72] Tziortzi , A. C. , Searle , G. E. , Tzimopoulou , S. , Salinas , C. , Beaver , J. D. , Jenkinson , M. , Laruelle , M. , Rabiner , E. A. , & Gunn , R. N. ( 2011 ). Imaging dopamine receptors in humans with [11C]-(+)-PHNO: Dissection of D3 signal and anatomy . Neuroimage , 54 ( 1 ), 264 – 277 . 10.1016/j.neuroimage.2010.06.044 20600980

[b73] Velázquez-Zamora , D. A. , Garcia-Segura , L. M. , & González-Burgos , I. ( 2012 ). Effects of selective estrogen receptor modulators on allocentric working memory performance and on dendritic spines in medial prefrontal cortex pyramidal neurons of ovariectomized rats . Hormones and Behavior , 61 ( 4 ), 512 – 517 . 10.1016/j.yhbeh.2012.01.010 22285935

[b74] Viswanathan , A. , & Freeman , R. D. ( 2007 ). Neurometabolic coupling in cerebral cortex reflects synaptic more than spiking activity . Nature Neuroscience , 10 ( 10 ), 1308 – 1312 . 10.1038/nn1977 17828254

[b75] Wang , H. , Xu , X. , Xu , X. , Gao , J. , & Zhang , T. ( 2020 ). Enriched environment and social isolation affect cognition ability via altering excitatory and inhibitory synaptic density in mice hippocampus . Neurochemical Research , 45 , 2417 – 2432 . 10.1007/s11064-020-03102-2 32748366

[b76] Wylie , G. , & Allport , A. ( 2000 ). Task switching and the measurement of “switch costs” . Psychological research , 63 ( 3 ), 212 – 233 . 10.1007/s004269900003 11004877

[b77] Yarkoni , T. , Poldrack , R. A. , Nichols , T. E. , Van Essen , D. C. , & Wager , T. D. ( 2011 ). Large-scale automated synthesis of human functional neuroimaging data . Nature Methods , 8 ( 8 ), 665 – 670 . 10.1038/nmeth.1635 21706013 PMC3146590

