# Supplementary material for: The relationship between SV2A levels, neural activity, and cognitive function in healthy humans: A [11C]UCB-J PET and fMRI study
Source: Imaging Neurosci (Camb). 2024 Jun 10;2:imag-2-00190. doi: 10.1162/imag_a_00190 (PMC11840333; doi:10.1162/imag_a_00190)
Supplement: Supplementary Material [file imag_a_00190-supp.pdf]

Supplementary materials

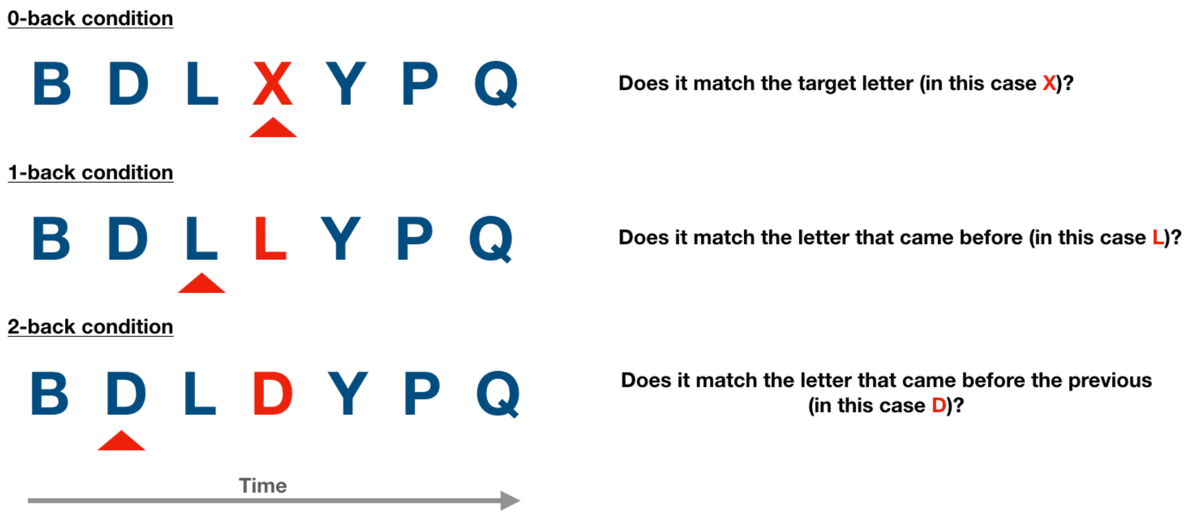

Supplementary figure 1: N-Back working memory task. The task consisted of 0-back, 1-back and 2-back blocks. During the 0-back block, participants were required to remember an initial target letter (shown in red) and identify whether the subsequent letters match the target. During the 1 and 2-back blocks, the participants had to recall whether the subsequent letters matched the letter presented one or two trials prior, respectively (indicated with red arrow).

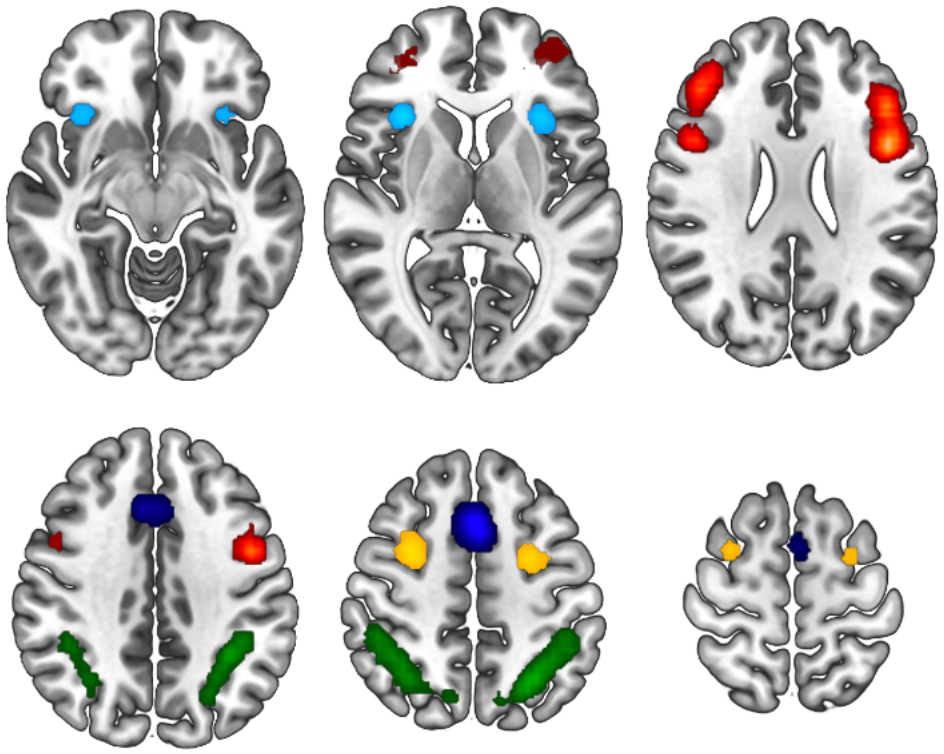

Supplementary figure 2: N-back working memory task *a priori* selected ROIs: Prefrontal cortex (maroon), dorsolateral prefrontal cortex (red), insula (light blue), parietal cortex (green), posterior fronal cortex (yellow), anterior cingulate cortex (dark blue). Shown in MNI152 space, slices are: A -10 7 25; 38 50 62

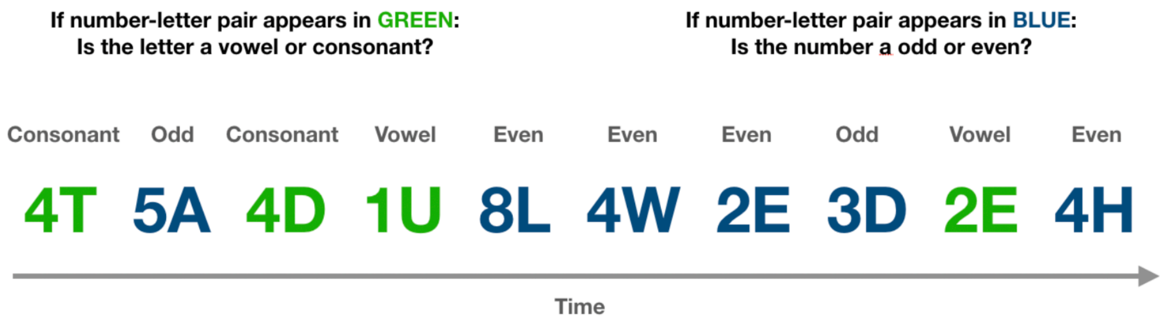

Supplementary figure 3: Task switching executive function task. Pairs of numbers and letters appeared on the screen in blue or green. If the number-letter pair appeared in green, participants were required to focus on the letter and respond if the letter is a vowel or consonant using an MRI-compatible response box. If the number-letter pair appeared in blue, participants were required to focus on the number and response if it is odd or even.

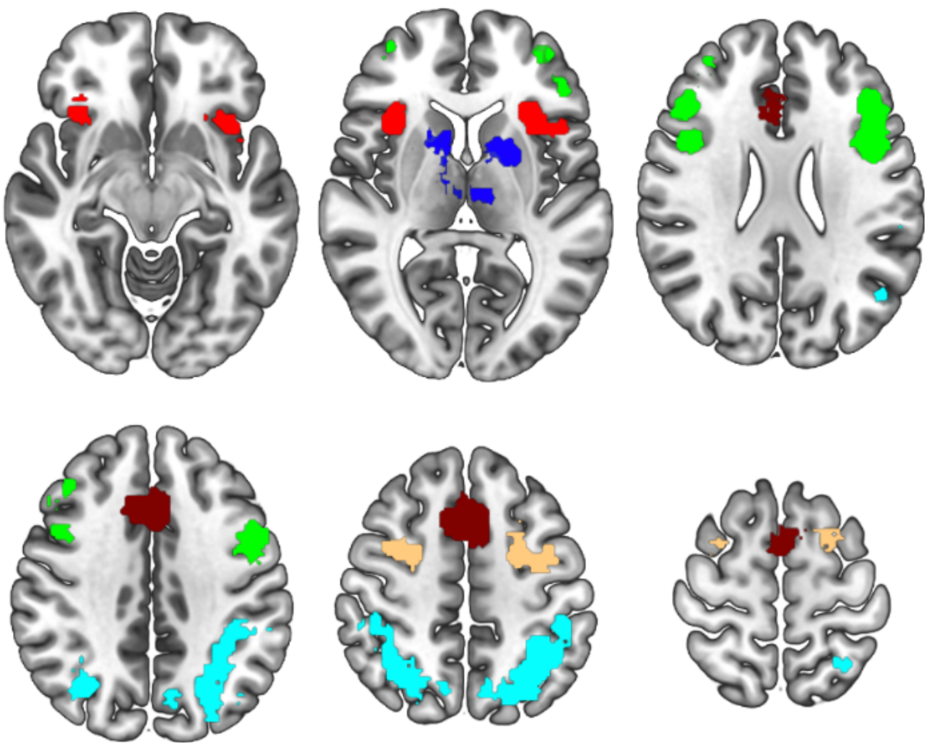

Supplementary figure 4: Task switching *apriori* selected ROIs: Dorsolateral prefrontal cortex (green), insula (red), parietal cortex (light blue), posterior frontal cortex (beige), anterior cingulate cortex (maroon), thalamus-putamen (dark blue). Shown in MNI152 space, slices are: A -10 7 25; 38 50 62

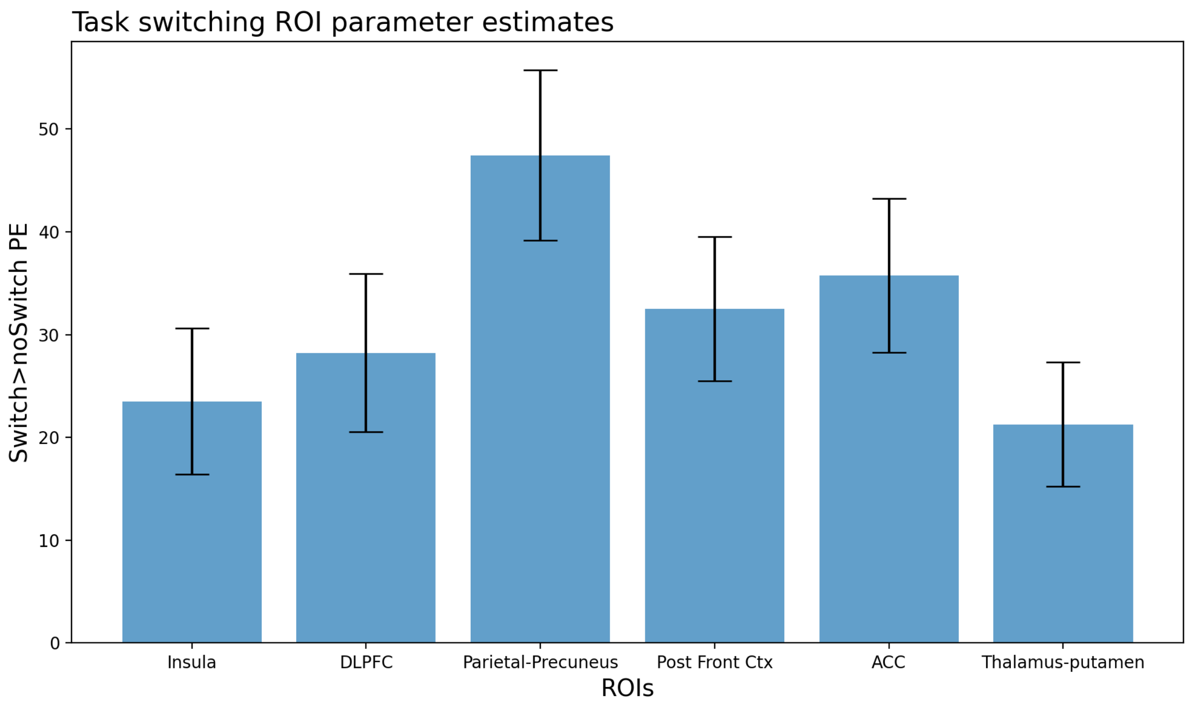

Supplementary figure 5: Engagement of task switching ROIs during the switch>no-switch condition. PE – Parameter Estimates.

Supplementary Table 1: Canonical weights for [11C]UCB-J PET and task switching fMRI data included in the PLS-CA analysis

		Insula	Dorsolateral prefronal cortex	Parietal-precuneus	Posterior frontal Cortex	Anterior Cingulate Cortex	Thalamus-putamen
First component	PET	0.353	0.335	0.544	0.521	0.380	0.225
	fMRI	0.646	0.216	0.015	-0.397	0.033	0.614
Second component	PET	-0.327	-0.095	0.486	-0.281	-0.328	0.679
	fMRI	-0.313	-0.441	-0.496	-0.603	-0.289	0.122

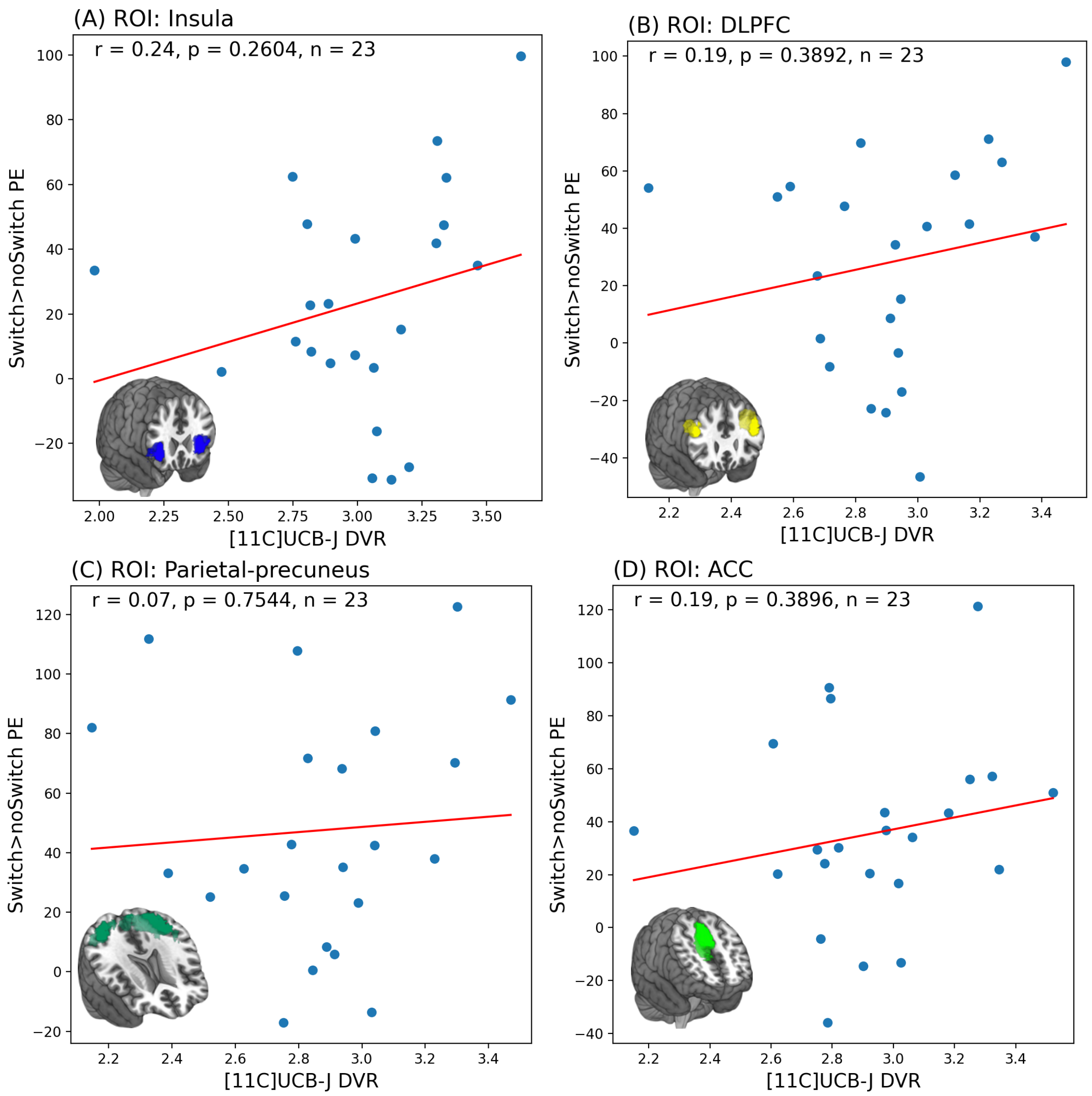

Supplementary figure 6: Additional exploratory univariate analyses (Pearson's correlation) testing relationship between [11C]UCB-J DVRcs and switch>no-switch parameter estimates in the (A) Insula, (B) Dorsolateral prefrontal cortex (DLPFC), (C) Parietal-precuneus and (D) anterior cingulate cortex (ACC) ROIs.

Supplementary Table 2: Weights of the contributions of each [11C]UCB-J DVRcs values for each ROI for each of the two components for the PLS-regression.

	Insula	Dorsolateral prefronal cortex	Parietal-precuneus	Posterior frontal Cortex	Anterior Cingulate Cortex	Thalamus-putamen
PLS weights for PET (first component)	0.374	0.430	0.487	0.460	0.351	0.321
PLS weights for PET (second component)	0.288	-0.072	-0.525	-0.348	0.468	0.544

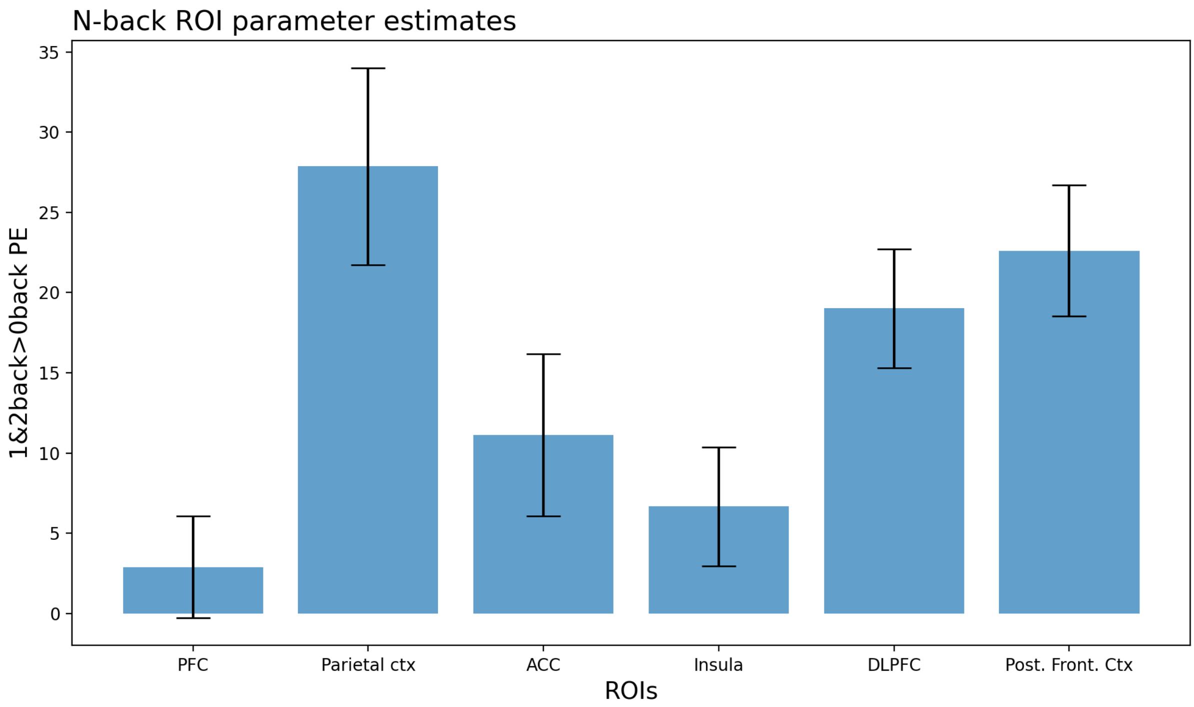

Supplementary figure 7: Engagement of N-back ROIs during the 1&2back>0back condition.

Supplementary Table 3: Canonical weights for [11C]UCB-J PET and n-back fMRI data included in the PLS-CA analysis

		Prefrontal cortex	Parietal cortex	Anterior Cingulate Cortex	Insula	Dorsolateral prefronal cortex	Posterior frontal Cortex
First component	PET	0.191	0.546	0.521	0.193	0.288	0.523
	fMRI	-0.293	-0.342	-0.424	-0.667	-0.353	-0.218
Second component	PET	0.724	-0.301	0.101	0.547	0.035	-0.273
	fMRI	0.401	-0.409	-0.134	0.229	0.245	-0.736

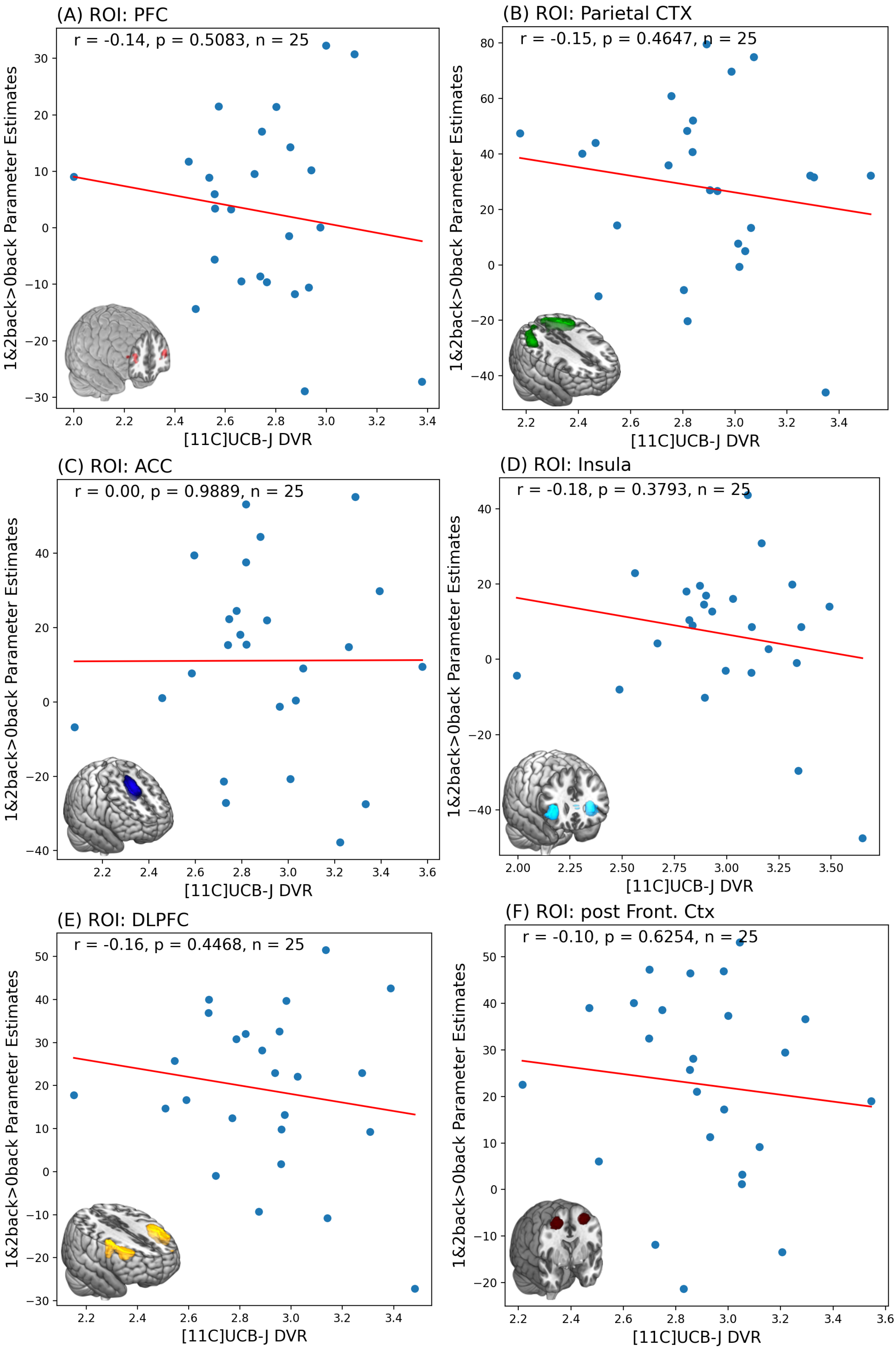

(A) ROI: PFC
r = -0.14, p = 0.5083, n = 25
(B) ROI: Parietal CTX
r = -0.15, p = 0.4647, n = 25
(C) ROI: ACC
r = 0.00, p = 0.9889, n = 25
(D) ROI: Insula
r = -0.18, p = 0.3793, n = 25
(E) ROI: DLPFC
r = -0.16, p = 0.4468, n = 25
(F) ROI: post Front. Ctx
r = -0.10, p = 0.6254, n = 25
1&2back>0back Parameter Estimates
[11C]UCB-J DVR

Supplementary figure 8: Exploratory univariate analyses showing lack of relationships between [11C]UCB-J DVR and task induced working memory related neural activity (1&2back>0back parameter estimates) in the (A) PFC, (B) parietal cortex, (C) ACC, (D) Insula, (E) DLPFC, (F) posterior frontal cortex.

Supplementary Table 4: Weights of the contributions of each [11C]UCB-J DVRcs values for each ROI for each of the two components for the PLS-regression that evaluated the relationship between N-back working memory scores and [11C]UCB-J DVRcs.

	Prefrontal cortex	Parietal cortex	Anterior Cingulate Cortex	Insula	Dorsolateral prefronal cortex	Posterior frontal Cortex
PLS weights for PET (first component)	0.387	0.407	0.450	0.368	0.413	0.420
PLS weights for PET second component)	-0.273	-0.045	0.700	-0.617	-0.116	0.198

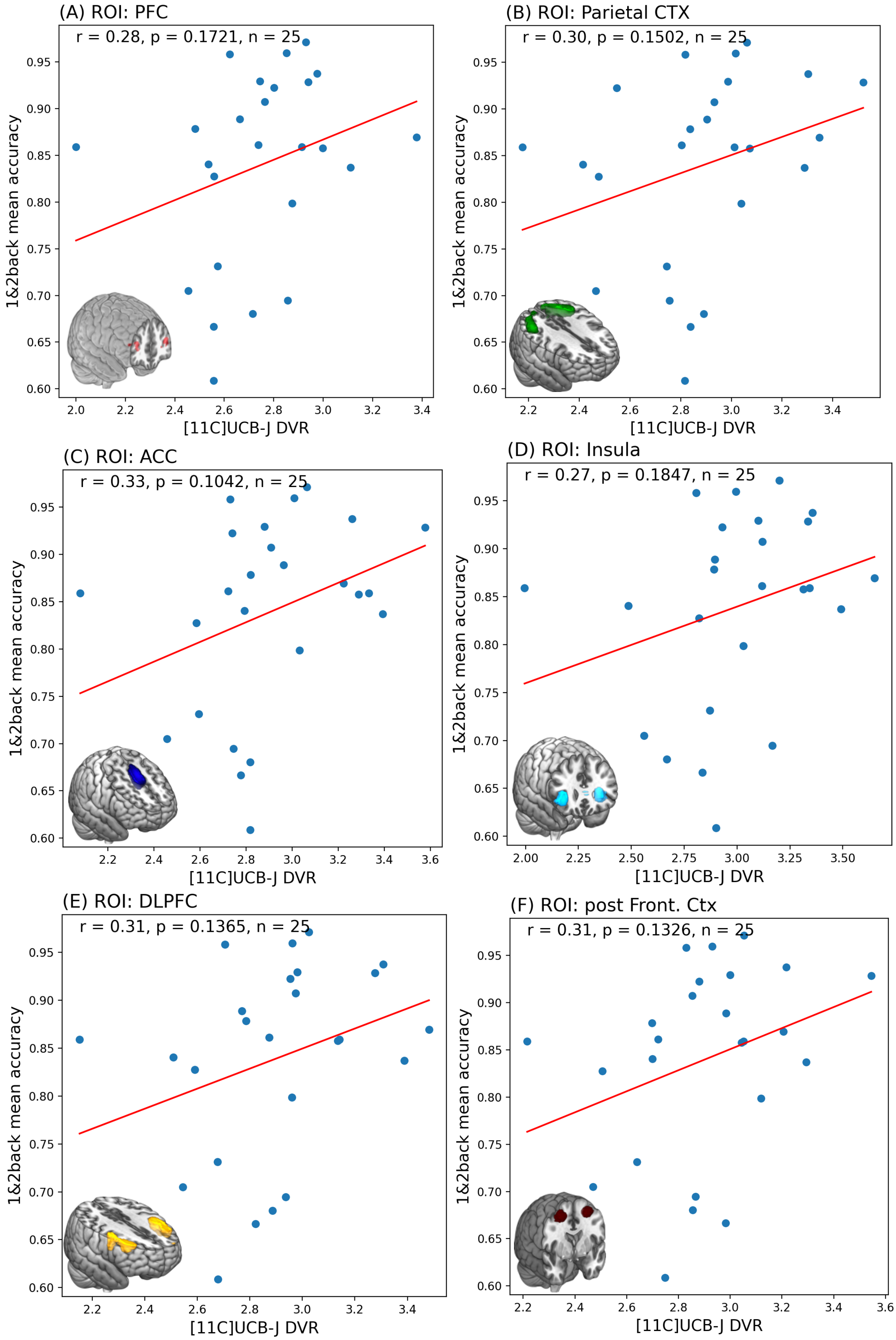

(A) ROI: PFC
r = 0.28, p = 0.1721, n = 25
1&2back mean accuracy
[11C]UCB-J DVR
(B) ROI: Parietal CTX
r = 0.30, p = 0.1502, n = 25
1&2back mean accuracy
[11C]UCB-J DVR
(C) ROI: ACC
r = 0.33, p = 0.1042, n = 25
1&2back mean accuracy
[11C]UCB-J DVR
(D) ROI: Insula
r = 0.27, p = 0.1847, n = 25
1&2back mean accuracy
[11C]UCB-J DVR
(E) ROI: DLPFC
r = 0.31, p = 0.1365, n = 25
1&2back mean accuracy
[11C]UCB-J DVR
(F) ROI: post Front. Ctx
r = 0.31, p = 0.1326, n = 25
1&2back mean accuracy
[11C]UCB-J DVR

Supplementary figure 9: Exploratory univariate analyses showing lack of relationships between [11C]UCB-J DVR and mean 1&2back accuracy for the N-back working memory task in (A) PFC, (B) parietal cortex, (C) ACC, (D) Insula, (E) DLPFC, (F) posterior frontal cortex.

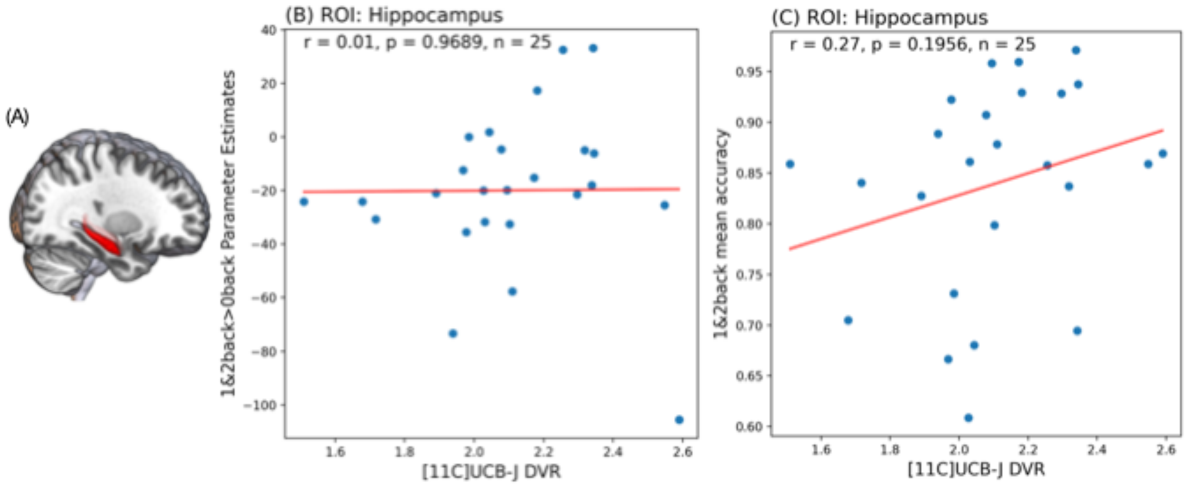

Supplementary figure 10: Exploratory univariate analyses showing lack of relationships between [11C]UCB-J DVR and N-back 1&2back>0back parameter estimates and mean N-back 1&2back accuracy in the hippocampus (A) hippocampus mask (B) Pearson's correlation between [11C]UCB-J DVR and N-back 1&2back>0back parameter estimates (C) Pearson's correlation between [11C]UCB-J DVR and mean N-back 1&2back accuracy.

Supplementary table 5: Variability of [11C]UCB-J DVRcs across regions of interest used for task switching and the n-back working memory task.

Task	ROI Name	Mean	Standard Deviation	Variance	Range	Coefficient of Variation
Task Switching	Insula	3.010	0.350	0.122	1.650	0.116
Task Switching	Dorsolateral prefrontal cortex	2.914	0.295	0.087	1.343	0.101
Task Switching	Parietal-precuneus	2.862	0.319	0.101	1.322	0.111
Task Switching	Posterior Frontal Cortex	2.873	0.278	0.077	1.311	0.097
Task Switching	Anterior cingulate cortex	2.940	0.298	0.089	1.370	0.101
Task Switching	Thalamus-putamen	2.512	0.336	0.113	1.376	0.134
N-back	Prefrontal cortex	2.744	0.266	0.071	1.377	0.097
N-back	Parietal cortex	2.883	0.311	0.097	1.344	0.108
N-back	Anterior cingulate cortex	2.905	0.326	0.106	1.496	0.112
N-back	Insula	2.995	0.351	0.123	1.654	0.117
N-back	Dorsolateral prefrontal cortex	2.901	0.299	0.089	1.330	0.103
N-back	Posterior Frontal Cortex	2.896	0.283	0.080	1.329	0.098
